# Synergistic inhibition of NUDT21 by secretory S100A11 and exosomal miR‐487a‐5p promotes melanoma oligo‐ to poly‐metastatic progression

**DOI:** 10.1002/1878-0261.13480

**Published:** 2023-07-01

**Authors:** Bin Zeng, Yuting Chen, Hao Chen, Qiting Zhao, Zhiwei Sun, Doudou Liu, Xiaoshuang Li, Yuhan Zhang, Jianyu Wang, H. Rosie Xing

**Affiliations:** ^1^ Institute of Life Sciences Chongqing Medical University China; ^2^ State Key Laboratory of Ultrasound in Medicine and Engineering, College of Biomedical Engineering Chongqing Medical University China

**Keywords:** exosome, glycolysis, miR‐487a‐5p, Nudt21, S100A11, Sec23a

## Abstract

Although early diagnosis and therapeutic advances have transformed the living quality and outcome of cancer patients, the poor prognosis for metastatic patients has not been significantly improved. Mechanisms underlying the complexity of metastasis cannot be simply determined by the straightforward ‘cause‐and‐effect relationships’. We have developed a ‘dry‐lab‐driven knowledge discovery and wet‐lab validation’ approach to embrace the complexity of cancer and metastasis. We have revealed for the first time that polymetastatic (POL) melanoma cells can utilize both the secretory protein pathway (S100A11–Sec23a) and the exosomal crosstalk (miR‐487a‐5p) to transfer their ‘polymetastatic competency’ to the oligometastatic (OL) melanoma cells, via synergistic co‐targeting of the tumor‐suppressor Nudt21. The downstream deregulated glycolysis was verified to regulate metastatic colonization efficiency. Further, two gene sets conferring independent prognosis in melanoma were identified, which have the potential for clinical translation and merit future clinical validation.

## Graphical abstract

The metastatic microenvironment involves cooperation between cancer and noncancer cells. This study focused on secretory protein (S100A11–Sec23a) and exosomal (miR‐487a‐5p) pathways in OL and POL melanoma cells. POL cells transfer polymetastatic competency to OL cells, which switch phenotype. Nudt21 targeting relieves the inhibition of glycolysis. Gene sets aid prognosis, showcasing the approach's clinical relevance.

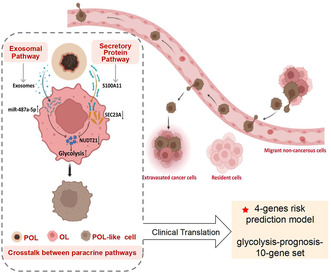

**Abbreviations**
AUC: area under ROC curve
CM: conditioned medium
GEO: Gene Expression Omnibus
GFP: green fluorescent proteins
GO: Gene Ontology
GSEA: gene set enrichment analysis
HR: hazard ratio
KEGG: Kyoto Encyclopedia of Genes and Genomes
LASSO: least absolute shrinkage and selection operator
MME: metastatic microenvironment
MSigDB: molecular signatures database
NES: normalized enrichment fraction
OL: oligometastatic cell line
POL: polymetastatic cell line
TCGA: The Cancer Genome Atlas
TME: tumor microenvironment

## 1 Introduction

Cancer progression requires the establishment of a dynamic tumor microenvironment (TME) [1, 2, 3]. The importance of metastatic microenvironment (MME) in metastasis has been increasingly appreciated [4, 5]. MME formation at the distant organ is analog to the process of ‘an orchestra in the making’. Resident cells of the distant organ, newly extravasated cancer cells, and migrant noncancerous cells (vascular endothelial cells, fibroblasts, various types of immune cells, etc.) become the new habitats and the builders of the MME. The intricate communications between heterogeneous cancer cells in the MME can utilize the crosstalk between the two paracrine pathways—the secretory protein pathway and the exosomal transport.

In TME or MME, cancer cells communicate with one another, or with stromal cells by two paracrine pathways [6, 7]: (a) secretory proteins that include growth factors, chemokines, and inflammatory factors [8, 9, 10] and (b) exosomes that encapsulate biologically active constituents that include but not limited to RNAs, DNAs, and proteins [11, 12, 13]. However, due to the experimental limitations, these two pathways have been studied separately for the most part and their crosstalk in the context of TME or MME has not been examined in‐depth at the mechanistic level [14, 15]. While interactions among different types of cells in TME or MME have gained an increasing appreciation, the majority of the efforts have been devoted to elucidating the communications between cancerous and noncancerous cells (such as fibroblasts, vascular endothelial cells, immune cells) [11, 12, 13]. Less attention has been paid to the interactions among diverse cancer cells. Thus, less is known about the contribution of such interactions to tumor progression and to the development of heterogeneity.

Here, we report the crosstalk between the secretory protein pathway and the exosomal pathway in facilitating the interactions between heterogeneous melanoma cancer cells. The stable and homologous oligometastasis (OL) and polymetastasis (POL) cell models previously established by our research group were utilized to simulate two heterogeneous cancer cell subsets with different metastatic colonization efficiency [16]. In the present study, they were used as low‐metastatic and high‐metastatic models, respectively. We have simulated the synergistic mechanisms exploited by OL and POL cells in the MME to facilitate their optimal interactions. We have revealed for the first time that the polymetastatic POL cells can utilize both the secretory protein pathway (S100A11‐Sec23a) and the exosomal crosstalk (miR‐487a‐5p) synergistically to transfer their ‘polymetastatic competency’ to the oligometastatic OL cells. As a result, the *in vivo* course of OL cells has been switched from oligo‐ to polymetastasis. This is achieved via synergistic co‐targeting of the tumor‐suppressor activity of Nudt21 by secretory ‘S100A11‐Sec23a’ and exosomal miR‐487a‐5p in OL cells. Thus, we have characterized a new molecular mechanism underlying oligo‐ to polymetastatic progression. In addition, we have identified and verified deregulated glycolysis as one of the metabolic changes that may regulate metastatic colonization efficiency. Furthermore, we have identified 2 gene sets conferring independent prognostic significance for melanoma that merits future clinical validation.

## 2 Materials and methods

### 2.1 Wet‐lab materials and methods

#### 2.1.1 Cell lines and cell culture

A375 cell (human SKCM cells, RRID: CVCL_0132) was purchased from the Chinese Academy of Sciences Cell Bank (Shanghai, China). GFP‐labeled Mouse melanoma M14 cells, labeled with green fluorescent proteins (GFP) were obtained from Dr. Robert Hoffman (University of California San Diego). Oligometastatic cell line (OL) and polymetastatic cell line (POL) were previously constructed and characterized by our research group—we had performed 3 rounds of *in vivo* generation: (a) generate the paired lung derivative melanoma cell lines; (b) generate the distinct oligo‐ and polymetastatic lung cell lines and (c) confirm the stability of oligo‐ and polymetastatic progression [17, 18, 19]. Cells were cultured at 37 °C with 5% CO_2_ in DMEM medium (Gibco, Grand Island, NY, USA) prepared with 10% fetal bovine serum (FBS, Gibco, Grand Island, NY, USA) and 1% penicillin–streptomycin (Hyclone, Logan, UT, USA).

All cell lines used in this study have been authenticated within the past 3 years. Cell line authentication involves DNA profiling to verify identity and detect contamination or misidentification. Our study followed established protocols, including STR analysis, to ensure accurate cell line identification and reliability of results. Experiments were performed using mycoplasma‐free cells. We conducted regular screenings using sensitive methods such as PCR to confirm the absence of mycoplasma in our cell cultures, ensuring reliable results.

#### 2.1.2 Lentiviral infections and cell transfection

The lentivirus packaging services for overexpression and knockdown of Sec23a were provided by Shanghai Sangon Biotech Co., Ltd (Shanghai, China). Lentiviruses overexpressing Nudt21 and miRNA‐487a‐5p were purchased from Shanghai Genechem Co., Ltd (Shanghai, China).

The control and sequence‐specific mimics and inhibitor of miRNA‐487a‐5p, and the Sparc‐knockdown siRNA (Si‐Sparc) were purchased from Shanghai GenePharma Co., Ltd (Shanghai, China). Lipofectamine 2000 agents (Invitrogen, Grand Island, NY, USA) were used for the transient infection of miRNA‐487a‐5p‐mimics, miRNA‐487a‐5p‐inhibitor, and Si‐Sparc according to the manufacturer's instructions.

#### 2.1.3 Reverse transcription and quantitative real‐time polymerase chain reaction (RT‐qPCR)

Total RNA of cells was prepared using Trizol (Cowin Bio., Beijing, China). The purity and concentration of RNA were detected by UV spectrophotometer. cDNA was synthesized by PrimeScript RT Master Mix and Mir‐X miRNA First‐Strand Synthesis Kit (Takara, Otsu, Japan), and further amplified by TB Green Premix Ex Taq II and Mir‐X miRNA qRT‐PCR TB Green Kitt. The steps of the reaction were set up as follows: (a) predenatured at 95 °C for 600 s, (b) 40 cycles of denaturation at 95 °C for 10 s, and (c) annealing at 60 °C for 30 s. The primer sequences were listed in Table S1.

#### 2.1.4 Western blotting analysis

Cells were harvested and cell pallets were treated with RIPA buffer (Beyotime, Shanghai, China) to extract whole‐cell proteins. The protein concentration was determined by the BCA method. The loading buffer was added to the aliquoted lysates and stored at −80 °C for future use. After boiling at 95 °C for 10 min, 40 ~ 50 μg protein of each sample were separated on 8%, 12%, or 15% polyacrylamide (Beyotime, Shanghai, China) gel for 1–2 h by electrophoresis and transferred to a PVDF membrane for 1 ~ 2 h. The membrane was first blocked by the QuickBlock™ Blocking buffer (Beyotime, P0252, Shanghai, China) for 10 ~ 15 min, then incubated with the primary antibody at 4 °C overnight. One‐hour incubation with the secondary antibody was performed at room temperature. The concentrations of the primary and secondary antibodies for western blotting followed the manufacturer's instructions. The membrane was developed in darkroom after being washed three times by 1× TBST. Primary antibodies used in the present study were as follows: anti‐SEC23A (CST, Cat No: 8162, Boston, MA, USA), anti‐Tubulin (CST, Cat No: 2146, Boston, MA, USA), anti‐S100A11 (Proteintech, Cat No: 10237‐1‐AP, Wuhan, China), anti‐SPARC (Proteintech, Cat No: 15274‐1‐AP, Wuhan, China), anti‐GAPDH (Proteintech, Cat No: 10494‐1‐AP, Wuhan, China), and anti‐NUDT21 (Proteintech, Cat No: 10322‐1‐AP, Wuhan, China).

#### 2.1.5 The co‐culture system

The Transwell chambers with 0.4 μm aperture in the 6‐well plate were used for the co‐culture experiments to simulate the indirect interactions between OL and POL cells. 5 × 10^5^ of POL and OL cells were inoculated into the upper chamber (POL) and the lower well (OL), respectively. Two‐liter culture medium were added to the respective OL and POL compartment. POL and OL cells were co‐cultured for 36 ~ 48 h.

#### 2.1.6 Mass spectrometry of secreted proteome

POL and OL cells were seeded in 5100‐mm culture plates, respectively, and allowed cells to grow to 50–70% confluent. Culture plates were washed with PBS. Cells were cultured in a serum‐free high glucose DMEM medium for additional 24 h. The conditioned medium was collected and centrifuged at 10 000 ***g*** for 1 h at 4 °C, and the cell‐free supernatant was stored at −80 °C for future analysis. LC–MS/MS detection and analysis were conducted by Shanghai Luming Biological Technology Co., Ltd (Shanghai, China). We downloaded the mass spectrometry measured values and performed *t*‐test analysis to compare the content of glycolytic intermediates in POL and OL cells.

#### 2.1.7 Exosomes preparation and characterization

Exosomes were isolated from the cell culture medium by sequential ultracentrifugation. Viable cells were seeded into the culture dish and let adhere to the growth surface overnight. Cells were maintained in a serum‐free medium for 36–48 h, and the conditioned medium (CM) was collected. CM was centrifuged by sequential and differential centrifugation: at 800, 2000, and 10 000 ***g*** for 10 min each. The resultant supernatant was filtrated and concentrated by the spin columns with 0.22 μm membrane. Afterwards, the concentrated supernatant was centrifuged at 4 °C and 100 000 ***g*** for 70 min. The sediment was collected and resuspended in PBS. The protein concentration of the exosomal preparations was determined by BCA protein determination kit (Pierce, Pierce County, WA, USA). Morphological features of exosomes were examined by transmission electron microscopy (JEM‐1400PLUS, JEOL, Beijing, China). The particle size of exosomes was analyzed by the nanoparticle tracking instrument (Nanosight‐NS300, Shanghai, China). The expression of exosome markers (CD63, CD81, and Alix) was examined by western blot.

#### 2.1.8 Exosome labeling and tracking analysis

OL‐exo was labeled with PKH26 fluorescent dye. The PKH26 fluorescent dye was adjusted to the working concentration and added to the exosome solution. The mixture was incubated at room temperature for 5–10 min, then co‐incubated with the same amount of 1% BSA for 1–2 min to terminate the staining reaction. The stained exosomes were collected and added to the culture medium of OL cells. After co‐incubation for 6 h, OL cells were washed with PBS and fixed with 70% paraformaldehyde for 30 min. Nuclei were stained with DAPI. Confocal fluorescence microscopy was used for visual observation of exosome labeling and tracking. Photos were taken.

#### 2.1.9 Exosomal miRNA sequencing and analysis

Exosomes from OL and POL cells were prepared as above and sent for sequencing. The construction, sequencing, and analysis of the miRNA library were completed by BGI. The OL and POL exosome samples have 3 replicate groups, respectively. We downloaded the raw sequencing data read count values and performed miRNA differential expression analysis using the R language ‘limma’ package to calculate LogFC (POL/OL) values and *P*‐values for the subsequent screening.

#### 2.1.10 Mass spectrometric analysis of metabolites

Glycolysis and TCA cycle metabolites in OL and POL cells were collected for capillary electrophoresis‐time‐of‐flight/mass spectrometry analysis. The detection and analysis were completed by Shanghai Applied Protein Technology Co., Ltd (Shanghai, China).

#### 2.1.11 Glucose, ATP, and lactic acid measurement

Key products of glycolysis, including glucose, ATP, and lactic acid, were quantified. Cell precipitates (more than 1 × 10^7^ cells) were collected for ultrasonic crushing. The glucose, ATP, and lactic acid concentrations were determined by microtitration assay using the Micro‐detection kit (Solarbio, Beijing, China) following the manufacturer's instructions.

#### 2.1.12 Transwell migration and invasion assays

The migration and Matrigel invasion assay utilized the 8 μm pore size Transwell inserts. A volume of 300‐μL cell suspension(3–5 × 10^5^ per mL) was added to the upper chamber of the Transwell, and a volume of 650 μL DMEM with 10% FBS was added to the lower chamber. After 12–16 h of incubation, the inserts were taken out and the nonmigrating or invading cells in the upper chamber of the Transwell were removed by a cotton swab. The chamber was rinsed and the cells were fixed with ice‐cold methanol solution for 15 min, followed by staining with Giemsa solution for 5–10 min. Finally, cells that migrated or invaded to the lower side of the membrane were observed. Five random visual fields were photographed under the inverted microscope and the migrated or invaded cells were counted.

#### 2.1.13 CCK8 proliferation assay

Cells were digested, resuspended, counted, and seeded into 96‐well plates. The density of inoculated cells was 1.5 × 10^3^ cells per well. After culturing the cells for 48 h, the cells to be tested were replaced with the fresh medium, and 10 μL of CCK8 reagent was added to each well and incubated for 2 h. The microplate reader detected the absorbance of each well at a wavelength of 450 nm and the survival rate was calculated.

#### 2.1.14 Luciferase reporter assay

First, the 3′ UTR sequence of Nudt21 gene was obtained from the gene bank, and the wild‐type fragment was constructed, containing the binding site of miRNA‐487a‐5p. The purified PCR product was cloned into psiCHECK‐2 vector and named as psiCHECK‐2‐NUDT21‐3′UTR. The construction of luciferase plasmid was completed by Hanbio Tech Co., Ltd (Shanghai, China). Two hundred ninety three T cells were plated and transfected with miRNA‐487a‐5p‐mimics, miRNA‐NC and the constructed Nudt21 luciferase vector when cell confluence reached 60–70%. After 48 h of the transfection, cells were lysed, and the luciferase activities of firefly and sea kidney were detected by the double luciferase assay.

#### 2.1.15 *In vivo* experiments

NOD/SCID mice, Male, 5 weeks old, were obtained from the Experimental Animal Centre of Chongqing Medical University (CQMU). NOD/SCID mice were housed in individually ventilated cages with suitable bedding and nesting materials, with a controlled temperature (20–26 °C) and humidity (40–60%). They were fed a balanced commercial rodent diet with access to clean drinking water. All *in vivo* experiments were conducted by trained personnel using aseptic techniques due to their compromised immune systems, in accordance with CQMU's t institutional animal welfare guidelines. 5 × 10^5^ OL or POL cells were injected via the tail vein to each mouse. Animals were sacrificed at the indicated time points. When mice were sacrificed, autopsies were performed for quantifying macroscopic metastases on the surface of the heart, lung, liver, kidney, and spleen.

#### 2.1.16 Ethical approval

All *in vivo* animal experiments received institutional approval from CQMU and were conducted in accordance with relevant guidelines and regulations (license number: SYXK2017‐0023), reviewed by Chongqing Medical University Laboratory Animal Management and Use Committee (IACUC‐CQMU).

#### 2.1.17 Statistical analysis

Statistical analysis was performed on experiments that had been performed at least three times. Quantitative results were presented as mean ± SD (standard error of the mean). The two‐tailed Student's *t*‐test in graphpad prism version 5.0 (GraphPad Software Inc., San Diego, CA, USA) was used for data analysis. *P* < 0.05, *P* < 0.01, and *P* < 0.001 were considered statistically significant and marked with single, double, and triple asterisk(s), respectively.

### 2.2 Bioinformatics analysis

#### 2.2.1 Data sources

GSE46517 melanoma microarray was obtained from the GEO database (Gene Expression Omnibus). This set contains 121 samples among which 31 were primary melanoma lesions, 73 were metastatic melanoma samples, 9 were nevus and 8 were normal skin samples, respectively. The raw count data of RNA and miRNA sequencing and relevant clinical information of human melanoma samples were extracted from TCGA database (The Cancer Genome Atlas). The RNA‐seq data were transformed into an expression matrix numbered with gene symbols, and then, the tumor tissues were grouped according to the expression of target genes of interest for further multidimensional analysis.

#### 2.2.2 GSEA analysis

Gene set enrichment analysis (GSEA) was performed. A total of 1000 random samples were arranged and the normalized enrichment fraction (NES) was compared. *P* < 0.05 with the false discovery rate (FDR) < 25% were regarded as statistically significant. Glycolytic genomes were obtained from the MSigDB (molecular signatures database). Gene expression profiles from GEO‐GSE46517 were used for the GESA analysis of glycolytic gene set in the primary and metastatic melanoma lesions.

#### 2.2.3 Gene expression, correlation, and survival analysis

Expression data of target genes were extracted from TCGA‐SKCM by the r ‘limma’ package. Pearson correlation test was performed to determine the correlations between gene expression. Survival analysis was performed by univariate and multivariate COX prognostic analysis through the r ‘survival’ package.

#### 2.2.4 LASSO machine learning for prioritization of target genes of miRNAs

Based on TCGA‐SKCM data, the prognosis of target genes was analyzed by univariate Cox regression analysis, and the predicted target genes with *P* < 0.2 were selected for subsequent LASSO (least absolute shrinkage and selection operator) analysis. The regression [20, 21] analysis package ‘glmnet’ based on LASSO from r software was used to identify predicted target genes that have clinical significance. The data of predicted target gene with *P* < 0.2 in the above univariate Cox analysis were included in LASSO disciplinary analysis model. The clinical risk prediction model was constructed for the prioritized target genes, and the area under the ROC curve (AUC), Kaplan–Meier analysis (product limit method), univariate, and multivariate Cox analysis were further carried out.

## 3 Results

### 3.1 POL cell‐derived exosomes augment the metastatic competency of OL cells

To ensure the reliability of the oligometastatic and polymetastatic phenotype of the OL and POL cells, respectively, we first verified that POL cells exhibited stronger invasiveness and colonization efficiency than that of the OL cells *in vitro* (Fig. S1A–D), which is consistent with our previous characterization [17, 19].

**Fig. 1:**
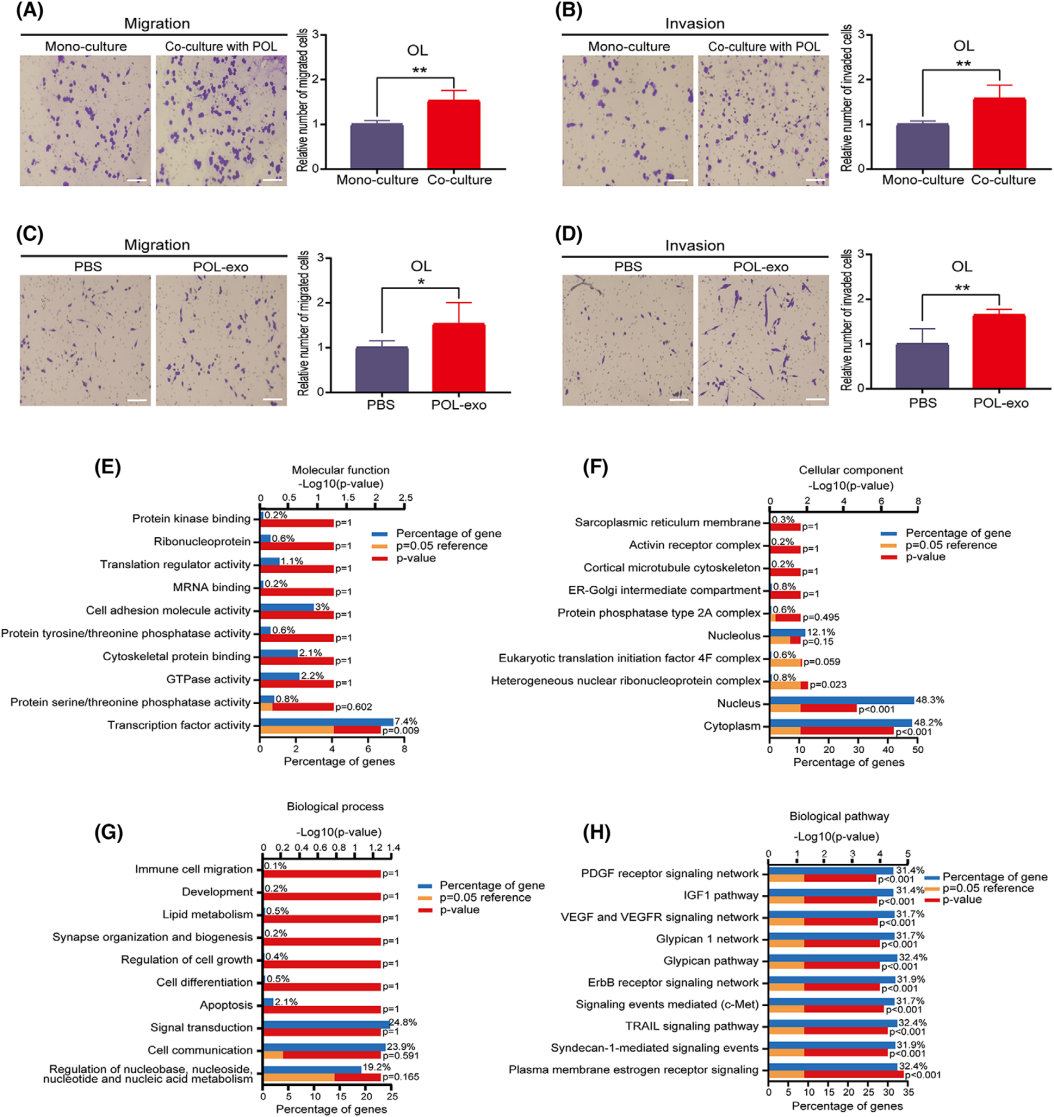
Analysis of differentially expressed miRNAs in POL and OL exosomes. (A, B) The OL cells that co‐cultured with POL cells exhibited stronger (A) migration and (B) invasion abilities compared with monocultured OL cells (bar = 60 μm); *n* = 3. (C, D) POL exosomes enhanced the (C) migration and (D) invasion abilities of OL cells (bar = 60 μm); *n* = 3. (E) Molecular function analysis of 25 exosomal DEMs. (F) Cellular component analysis of 25 exosomal DEMs. (G) Biological process analysis of 25 exosomal DEMs. (H) KEGG analysis of 25 exosomal DEMs. DEMs, differentially expressed miRNAs; OL, oligometastatic cell line; POL, polymetastatic cell line. *n* = 3: three times a particular experiment was replicated. Error bars indicated SD. *P < 0.05, **P < 0.01 by *t*‐test.

Next, we tested whether POL can modify the invasiveness of OL cells through paracrine interactions using the Transwell co‐culture system [22] (Section 2). Compared with OL cells in the monoculture, the invasiveness of co‐cultured OL cells was significantly enhanced (Fig. 1A,B). This observation indicates that substances released by POL cells into the culture media are capable of altering the metastatic competency of OL cells. We thus hypothesized that ‘POL cells can augment the metastatic competency of OL cells via secretory proteins or/and exosomal crosstalk’. To test this hypothesis, we first examined whether exosomal crosstalk is involved.

The exosomes of OL and POL were extracted and analyzed (Section 2) as we previously described [15]. We observed no significant differences in the morphology and size between exosomes derived from OL cells (OL‐exo) and from POL cells (POL‐exo) (Fig. S1E–G). POL‐exo or PBS were added to OL culture medium and let incubated for 24 h. Compared with the PBS‐added control group, OL cells treated with POL‐exo exhibited significantly enhanced invasiveness *in vitro* (Fig. 1C,D). These results show that POL cells can affect the metastatic competency of OL cells through exosomes.

### 3.2 Exosomal miRNA‐487a‐5p in POL‐exo augments the metastatic competency of OL cells *in vitro* and *in vivo*

The role of exosomal miRNAs in aiding the crosstalk between cancer and stromal cells in the TME or MME has been increasingly appreciated. By contrast, whether heterogeneous cancer cells can alter the inherent metastatic competency of one another; and if so, whether exosomal miRNAs facilitate such communications have not been investigated prior to our recent report [15].

**Fig. 2:**
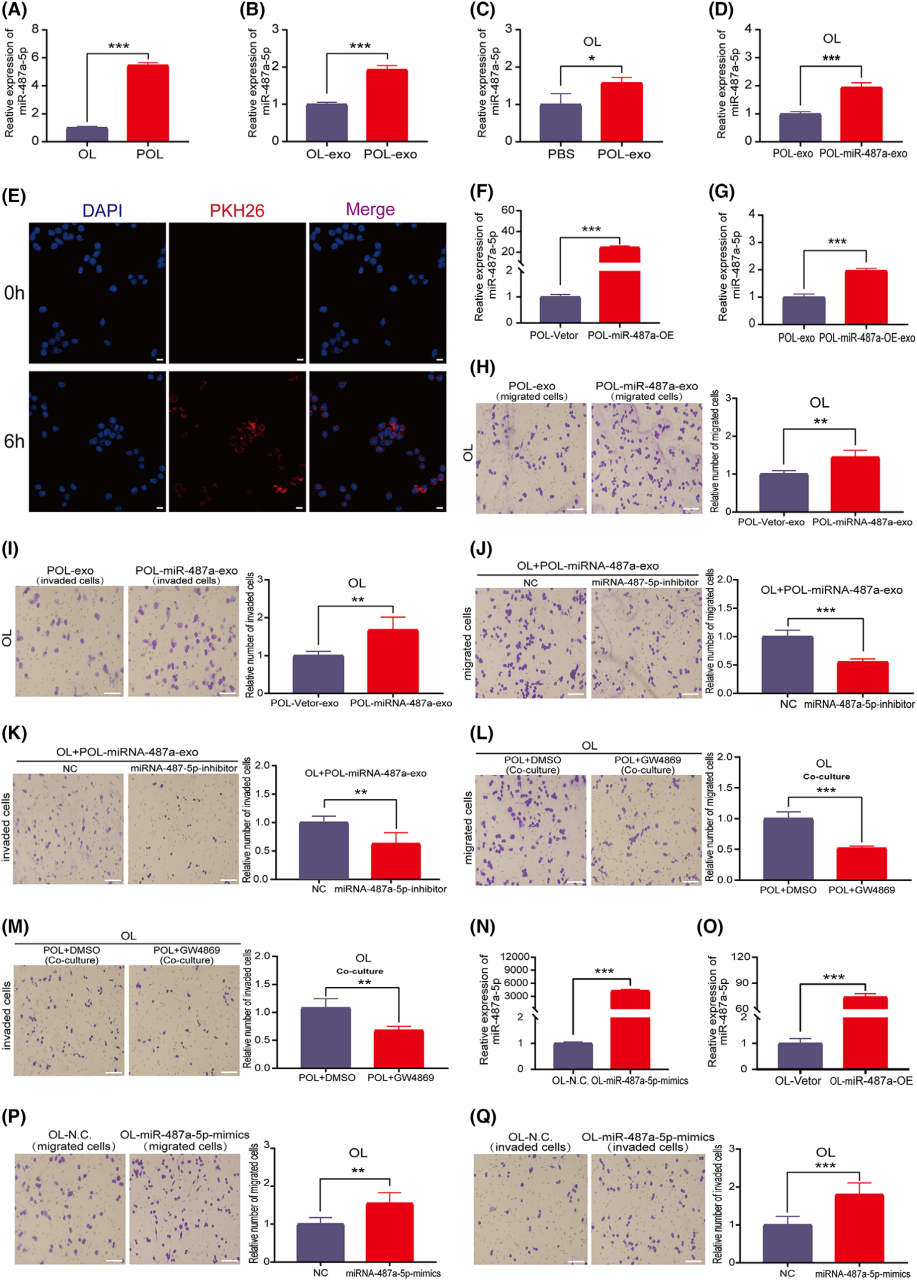
POL cells enhance the migration and invasion abilities of OL cells by delivering exosomal miRNA‐487a‐5p. (A, B) Expression differences of miRNA‐487a‐5p in OL and POL cells (A) and in their respective exosomes (B) detected by RT‐qPCR; *n* = 3. (C, D) miRNA‐487a‐5p expression in OL cells that were treated with POL exosomes (C), or treated with POL and POL‐miRNA‐487a‐OE exosomes (D). (E) Imaging changes of POL‐exo uptake by OL cells. POL cell‐derived exosomes were labeled with PKH26 (red); the nuclei of OL cells were labeled with DAPI (blue) (bar = 100 μm); *n* = 3. (F, G) Expression validation of miRNA‐487a‐5p in POL‐Vetor and POL‐miRNA‐487a‐OE cells (F) and in their respective exosomes (G); *n* = 3. (H, I) The POL‐miRNA‐487a‐OE exosomes promoted the (H) migration and (I) invasion abilities of OL cells (bar = 60 μm); *n* = 3. (J, K) miRNA‐487a‐5p‐inhibitor treatment reversed the increase in (J) migration and (K) invasion of OL cells that treated with POL‐miRNA‐487a‐OE exosomes (bar = 60 μm); *n* = 3. (L, M) GW6849 (20 μm) treatment reversed the increase in (L) migration and (M) invasion of OL cells that co‐cultured with POL cells (bar = 60 μm); *n* = 3. (N) Confirmation of the efficiency of miRNA‐487a‐5p‐mimic treatment on miRNA‐487a‐5p expression; *n* = 3. (O) Expression of miRNA‐487a‐5p in OL‐Vetor and OL‐miRNA‐487a‐OE cells; *n* = 3. (P, Q) miRNA‐487a‐5p‐mimic treatment enhanced the (P) migration and (Q) invasion abilities of OL cells (bar = 60 μm); *n* = 3. NC, negative control; OE, overexpression; OL, oligometastatic cell line; POL, polymetastatic cell line. *n* = 3: three times a particular experiment was replicated. Error bars indicated SD. *P < 0.05, **P < 0.01, ***P < 0.001 by *t*‐test.

High throughput sequencing was performed and analyzed (Section 2). Three hundred eighty‐one exosomal miRNAs were present in the POL‐exo and OL‐exo. Since we intend to elucidate the mechanisms underlying the pro‐metastatic activity of POL‐exo, we focused on the 93 miRNAs that showed higher expression in POL‐exo compared with OL‐exo, among which 25 miRNAs showed differential expression with Log (POL‐exo/OL‐exo) > 2 and *P* < 0.05 (Table 1). Gene Ontology (GO) and Kyoto Encyclopedia of Genes and Genomes (KEGG) functional analysis by Funrich software [23] found enrichment in a variety of intracellular signal transduction pathways (Fig. 1F–I). Through literature review, we selected 14 among the 25 differentially expressed miRNAs to verify their expression in OL and POL cells, as well as in OL‐exo and POL‐exo by RT‐qPCR. Compared with OL cells and OL‐exo, miRNA‐487a‐5p, and miRNA‐411‐5p were highly expressed in POL cells and POL‐exo, respectively (Fig. S2A,B, Fig. 2A,B). Since the function of miRNA‐487a‐5p in human cancer is poorly characterized, we prioritized it for mechanistic investigation.

**Table 1:** Differentially expressed miRNAs (DEMs) of POL and OL exosomes (Log FC(POL/OL) > 2 and *P* < 0.05).

Gene ID	LogFC (POL/OL)	*P* value (POL/OL)
miR‐4488	12.55074679	1.01E‐07
miR‐331‐3p	11.96578428	8.44E‐06
miR‐1296‐5p	11.38100211	9.51E‐04
miR‐1180‐3p	11.18797059	0.00217698
miR‐455‐3p	10.96578428	0.0050545
miR‐628‐3p	10.96578428	0.0050545
miR‐4516	10.96578428	0.0050545
miR‐106b‐3p	10.70303839	0.011932275
miR‐193a‐5p	10.70303839	0.011932275
miR‐151a‐3p	5.045837536	7.63E‐12
miR‐376c‐3p	4.624510361	1.95E‐26
miR‐412‐5p	4.45874324	3.95E‐15
miR‐1246	4.285546496	1.30E‐61
miR‐487a‐5p	4.08899112	1.35E‐06
miR‐383‐5p	3.857309575	3.79E‐12
miR‐1307‐3p	3.833250733	4.27E‐19
miR‐339‐5p	3.806582229	8.79E‐10
miR‐197‐3p	3.142985174	3.28E‐16
miR‐374c‐3p	2.930839334	1.37E‐33
miR‐493‐3p	2.737037727	1.98E‐06
miR‐6529‐5p	2.631528738	2.26E‐94
miR‐379‐3p	2.586405918	0.010598039
miR‐362‐5p	2.586405918	0.010598039
miR‐3120‐3p	2.321062651	6.71E‐04
miR‐411‐5p	2.284294169	0

First, we show that POL‐derived miRNA‐487a‐5p, upon entering OL cells through exosomal transfer can subsequently enhance the migration and invasion abilities of OL cells *in vitro* by the following evidence: (a) POL‐exo tracking experiment confirmed that miRNA‐487a‐5p in POL‐exo could be transferred to OL cells through the exosomal pathway and could enter OL cells (Fig. 2C–E). In addition, the overexpression of miRNA‐487a‐5p in POL cells leads to the elevation of miRNA‐487a‐5p in POL‐exo (Fig. 2F,G). (b) Treatment of OL cells with POL‐exo prepared from miRNA‐487a‐5p‐mimics treated POL cells augmented the *in vitro* invasiveness of OL cells (Fig. 2H,I). (c) Inhibition of exosomal secretion by GW6849, or reduction of miRNA‐487a‐5p content in POL‐exo by miRNA‐487a‐5p inhibitor effectively prevented the observed increase in OL cell invasiveness upon POL‐*exo* or miRNA‐487a‐5p‐mimics treatment, respectively (Fig. 2J–M). (d) The invasiveness (migration and invasion) of OL cells was significantly increased upon miRNA‐487a‐5p‐mimics treatment compared with that of control mimics treatment (Fig. 2N–Q).

**Fig. 3:**
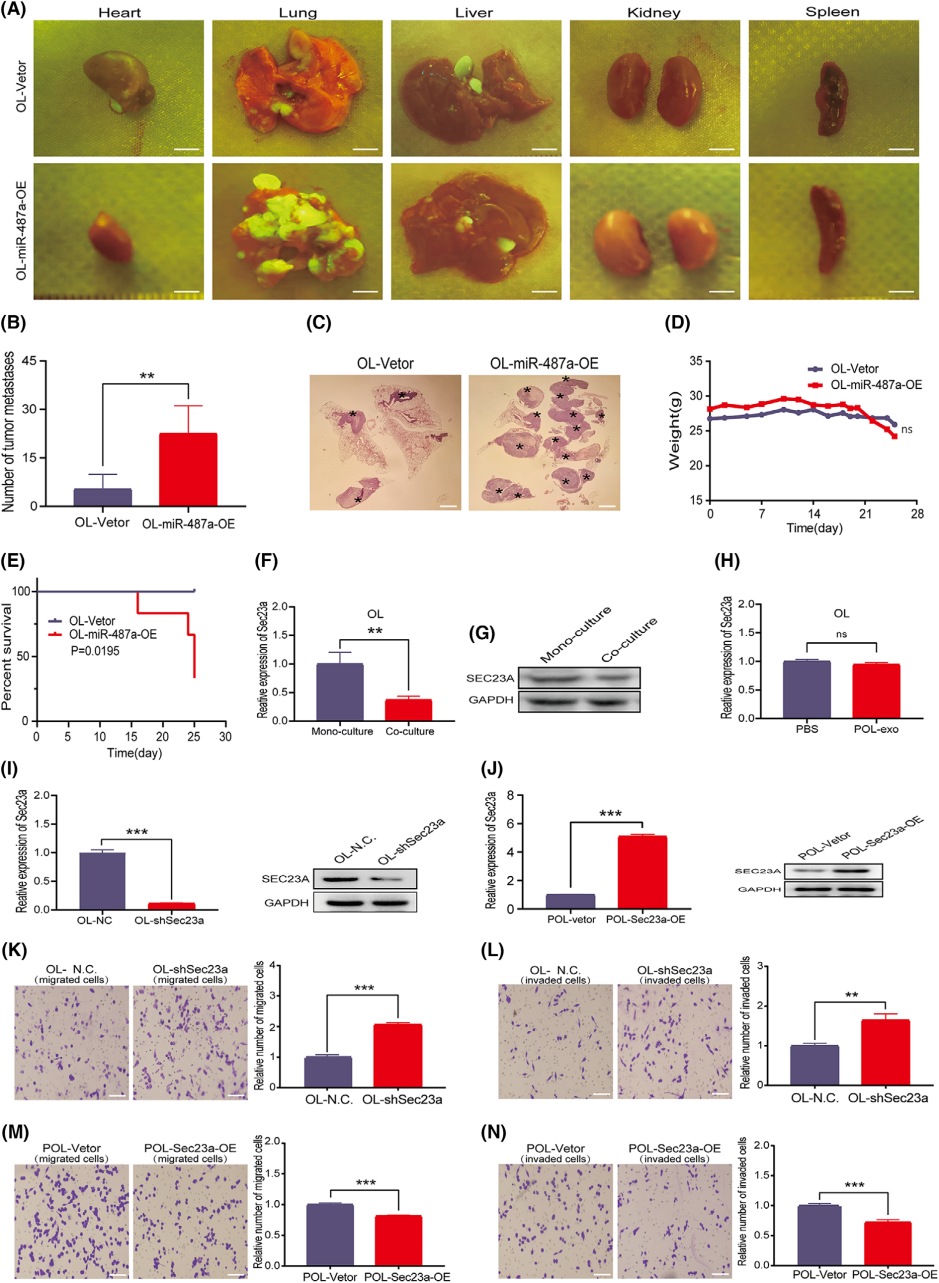
miRNA‐487a‐5p promotes melanoma metastasis *in vivo* and POL promotes metastasis of OL through secreted protein. (A) Photographic representation of macroscopic metastases of NOD/SCID mice 3 weeks after tail vein injection of control cells and overexpressed miRNA‐487a cells (OL‐Vetor and OL‐miRNA‐487a‐OE, bar = 3 mm); 5 mice per group. (B) Quantitative analysis of the superficial lung metastases of control and overexpressed miRNA‐487a cells. (C) Visualization of intrapulmonary metastases of control and overexpressed miRNA‐487a cells by H&E staining; Lung metastatic foci marked by*(bar = 2 mm). (D) Body weight changes of mice receiving control and overexpressed miRNA‐487a cells. (E) Survival analysis of control and miRNA‐487a‐OE groups within 30 days of tumor cell inoculation. (F, G) Expression of Sec23a in OL cells in monoculture and co‐culture; (F) RT‐qPCR; (G) WB; *n* = 3. (H) RT‐qPCR detection of Sec23a expression in OL cells treated with POL exosomes; *n* = 3. (I, J) Knockdown and overexpression efficiency of Sec23a; (left) PCR; (right) WB; *n* = 3. (K, L) Sec23a knockdown enhanced the (K) migration and (L) invasion abilities of OL cells (bar = 60 μm); *n* = 3. (M, N) Sec23a overexpression weakened the (M) migration and (N) invasive abilities of POL cells (bar = 60 μm); *n* = 3. POL, polymetastatic cell line; OE, overexpression; OL, oligometastatic cell line. *n* = 3: three times a particular experiment was replicated. Error bars indicated SD. *P < 0.05, **P < 0.01, ***P < 0.001 by *t*‐test.

Next, to evaluate the effect of miRNA‐487a‐5p on melanoma metastasis *in vivo*, OL cells that stably overexpressed miRNA‐487a‐5p (OL‐miRNA‐487a‐5p‐OE, Fig. 2O) were established by lentivirus infection. 5 × 10^5^ OL‐vector control or OL‐miRNA‐487a‐5p‐OE cells were injected into NOD/SCID mice through caudal vein, respectively. After 3 weeks of tumor cell inoculation, mice receiving OL‐miRNA‐487a‐OE cells developed multiorgan polymetastases (Fig. 3A), while the control group (OL‐vector injection) formed oligometastases in the lungs as we previously reported [16]. Quantification of the number of lung surface metastatic nodules confirmed that stable overexpression of miRNA‐487a‐5p in OL cells altered their *in vivo* phenotype from single‐organ oligometastasis to multiorgan polymetastasis (Fig. 3B). The number of lung metastases of miRNA‐487a‐5p overexpressed group was significantly more than those in the control group (Fig. 3C, Fig. S2C). Although no significant differences in the body weight of the mice were found in the two experimental groups (Fig. 3D), the mortality rate of the mice receiving OL‐miRNA‐487a‐OE cells was significantly higher than that in the control group (Fig. 3E). Collectively, these results show that miRNA‐487a‐5p promotes OL cells to undergo oligometastatic to polymetastatic progression *in vivo*.

Up to this point, we have verified the hypothesis that POL cells can augment the metastatic efficiency of OL cells via POL‐derived exosomal miRNA‐487a‐5p both *in vitro* and *in vivo*.

### 3.3 POL‐derived secretory proteins augment the *in vitro* invasiveness of OL cells by inhibiting Sec23a in OL cells

Next, we examined whether POL cells can augment the metastatic competency of OL cells via the secretory protein pathway. We have shown previously that Sec23a inhibits metastatic colonization efficiency of melanoma by regulating the transportation of secretory proteins [14, 24]. In this study, we first measured whether Sec23a mRNA and protein expression in OL cells were altered upon co‐culture with POL cells. Significant reduction of Sec23a mRNA and protein expression was found in OL cells co‐cultured with POL cells, compared with that in OL monoculture (Fig. 3F,G). Moreover, the involvement of POL‐exo in inhibiting Sec23a expression in OL cells was excluded (Fig. 3H). Therefore, it is most likely that secretory proteins in the medium of POL cell culture inhibit the expression of Sec23a in OL cells. Using the previously constructed OL‐shsec23a and POL‐Sec23a‐OE cells model (Fig. 3I,J), we verified the antimetastatic activity of Sec23a in OL cells (Fig. 3K–N).

So far, we tested our hypothesis that POL cells can augment the metastatic competency of OL cells via secretory proteins and the exosomal crosstalk. We left the identification and characterization of the secretory protein that inhibits Sec23a expression in OL cells to the last part of this study. We next focused on, at the mechanistic level, how POL cells can utilize the secretory protein pathway and exosomal crosstalk synergistically for the transfer of their ‘polymetastatic competency’ to OL cells.

### 3.4 Prioritization and validation of common mediators of miRNA‐487a‐5p and Sec23a

We analyzed the downstream target genes of Sec23a and miRNA‐487a‐5p to identify their common downstream mediators, with the aim of explaining the synergistic effect of secretory proteins and exosomes that underlies the pro‐metastatic activity of POL‐on OL cells. We employed the ‘dry‐lab discovery/wet‐lab validation’ approach to tackle synergistic interaction between the two paracrine pathways.

**Fig. 4:**
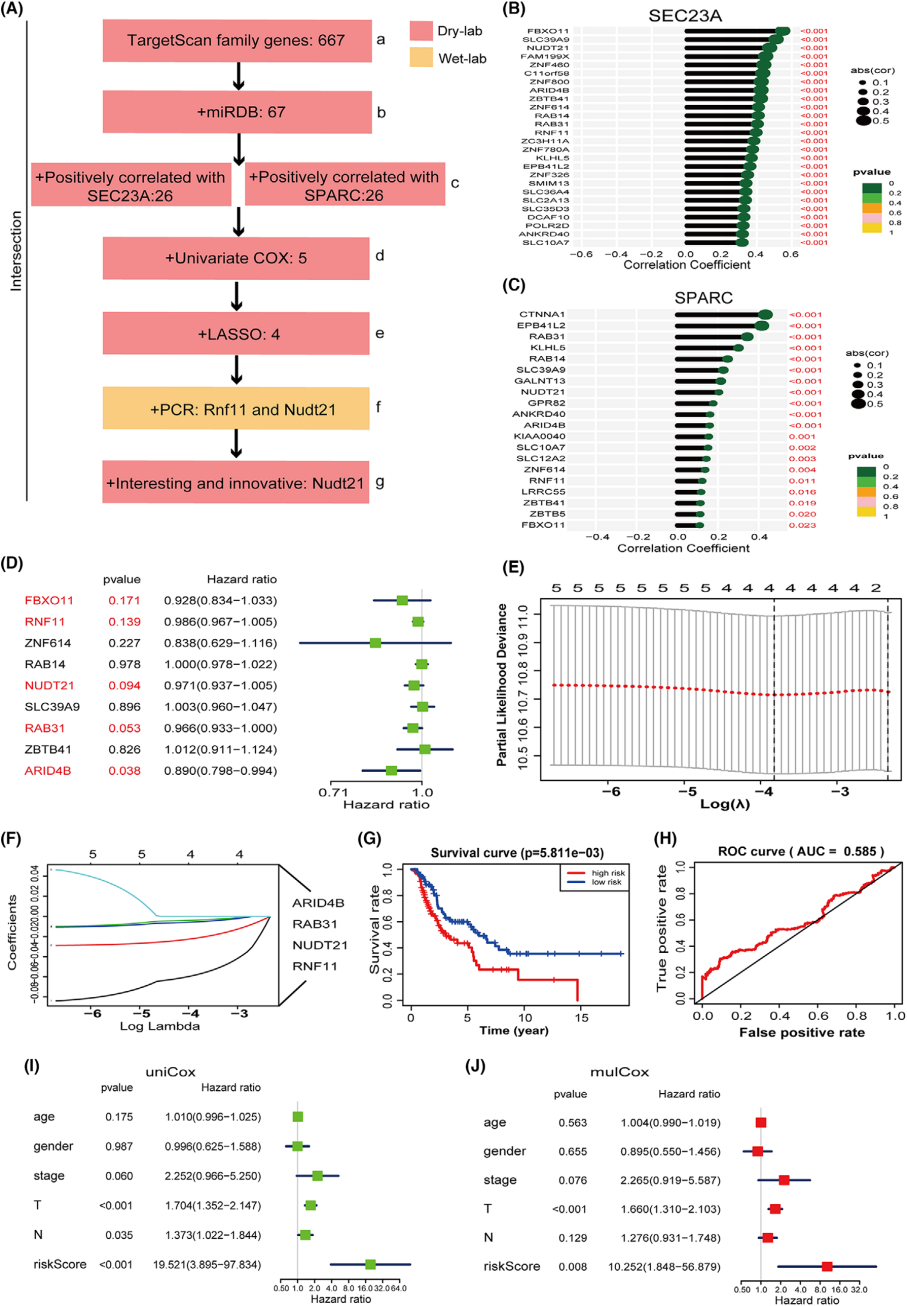
Nudt21 is a co‐regulated downstream gene of miRNA‐487a‐5p and SEC23A. (A) Schematic diagram of the miRNA‐487a‐5p target gene screening and prioritization. (B) 14 downstream genes that were positively correlated with Sec23a by Pearson correlation analysis. (C) 20 downstream genes that positively correlated with Sparc by Pearson correlation analysis. (D) Nine genes that were positively correlated with both Sec23a and Sparc were analyzed by univariate COX regression analysis using TCGA‐SKCM database; Error bars indicated 95% CI. (E, F) LASSO modeling. Five genes (*P* < 0.2, HR > 1) selected by univariate COX analysis were used for LASSO modeling (E), and finally, four genes including Rnf11, Nudt21, Rab31, and Arid4B were obtained (F). (G) Kaplan–Meier analysis of the risk model. (H) ROC curve analysis of the risk model. (I, J) Univariate (I) and multivariate (J) COX analyses of the risk model; Error bars indicated 95% CI. N, regional lymph node; TCGA, The Cancer Genome Atlas; T, tumor.

Target genes of miRNA‐487a‐5p were predicted using TargetScan, miRTarBase, and miRDB databases. We found that the number of the predicted gene targets varied considerably using the different databases (Fig. S3A). In order to improve the sensitivity and specificity of target gene prediction, 3038 TargetScan‐predicted target genes were grouped into 667 gene families, which were subsequently intersected with miRDB and yielded 67 candidate target genes for further analysis (Fig. 4Aa‐b). Since the co‐culture of OL cells with POL cells leads to Sec23a inhibition in OL cells, and Sec23a is antimetastatic in our prior characterization [14, 24], Sec23a‐dependent secretome was profiled by the protein mass spectrometry. We identified 28 downregulated secretory proteins in the conditioned medium of OL‐shSec23a cells in which Sec23a was silenced compared with that of OL cells (Fig. S3B).

We conducted correlation analysis between Sec23a and the ‘Sec23a‐dependent 28‐downregulated proteins’ using the tools provided by the GEPIA website. We found that Sparc and Sec23a had a relatively high positive correlation at the transcription level (*R* > 0.4) (Fig. S3C). PCR and WB analyses confirmed that Sparc mRNA and protein expression was subjected to Sec23a regulation in OL cells, respectively (Fig. S3D–G). Sparc knockdown in OL cells by siRNA‐Sparc significantly enhanced the *in vitro* invasiveness of OL cells (Fig. S3H,I). By contrast, the addition of recombinant protein rSPARC (8 μg·mL^−1^) significantly inhibited the *in vitro* invasiveness of OL‐shSec23a cells (Fig. S3J,K). Based on the above bioinformatics analysis and experimental validation, we confirmed that Sparc is a downstream mediator of Sec23a.

Next, Pearson correlation analysis combined with clinical evaluation using human melanoma data (TCGA‐SKCM) was performed to analyze the correlation between SEC23A, SPARC, and the 67 candidate gene targets of miR‐487a‐5p (Fig. 4Aa‐b). Pearson analysis identified 26 and 20 gene targets of miR‐487a‐5p were positively correlated with SEC23A (*R* > 0.3, *P* < 0.05) (Fig. 4B) and with SPARC (Fig. 4Ac,C), respectively. Clinical evaluation was conducted to further prioritize common targets of Sec23a and miR‐487a‐5p that have clinical significance. We utilized the patient data (177 patients) with lymph node and distant organs metastases from the TCGA‐SKCM data and identified 9 target genes (RAB31, RAB14, SLC39A9, NUDT21, ARID4B, ZNF614, RNF11, ZBTB41, FBXO11; Fig. 4Ac) that positively correlated with both SEC23A and SPARC. Univariate COX analysis revealed that RAB31, NUDT21, and ARID4B genes were tumor suppressive in clinical melanoma samples (*P* < 0.1; Fig. 4Ad). To avoid leaving out the valuable factors, 5 genes with *P* < 0.2 (Fig. 4D, red) were selected for LASSO modeling. Consequently, 4 genes (RNF11, NUDT21, RAB31, and ARID4B) were obtained (Fig. 4Ae,E,F) for constructing the ‘4‐genes risk prediction model’. Despite the relatively low AUC value, K‐M analysis, univariate and multivariate COX analysis all indicated that the risk prediction model conferred clinical prognosis (Fig. 4G–J). The hazard ratio (HR) of the ‘4‐genes risk prediction model’ was as high as 19.5 and 10.2 for the univariate and multivariate analysis, respectively, which was significantly higher than that of TNM (Fig. 4I,J). Thus, with the combination of miRNA databases, LASSO machine learning and clinical evaluation, 4 common target genes of Sec23a and miRNA‐487a‐5p in the Lasso risk prediction model, were prioritized.

In wet‐lab validation, the expression of Rnf11, Nudt21, Rab31, and Arid4b genes were verified by PCR in OL‐miR‐487a‐OE and OL‐shSec23a cells, respectively. Compared with OL cells, mRNA expression of Rnf11 and Nudt21 was significantly decreased in OL‐miR‐487a‐OE and OL‐shSec23a cells (Figs 4Af and 5A–D). Although the tumor‐suppressor function of NUDT21 has been reported in bladder cancer [25], breast cancer [26], and lung cancer [27, 28], its involvement in melanoma has not been reported preclinically and clinically. For the above considerations, Nudt21 gene was selected for further mechanistic investigation (Fig. 4Ag).

**Fig. 5:**
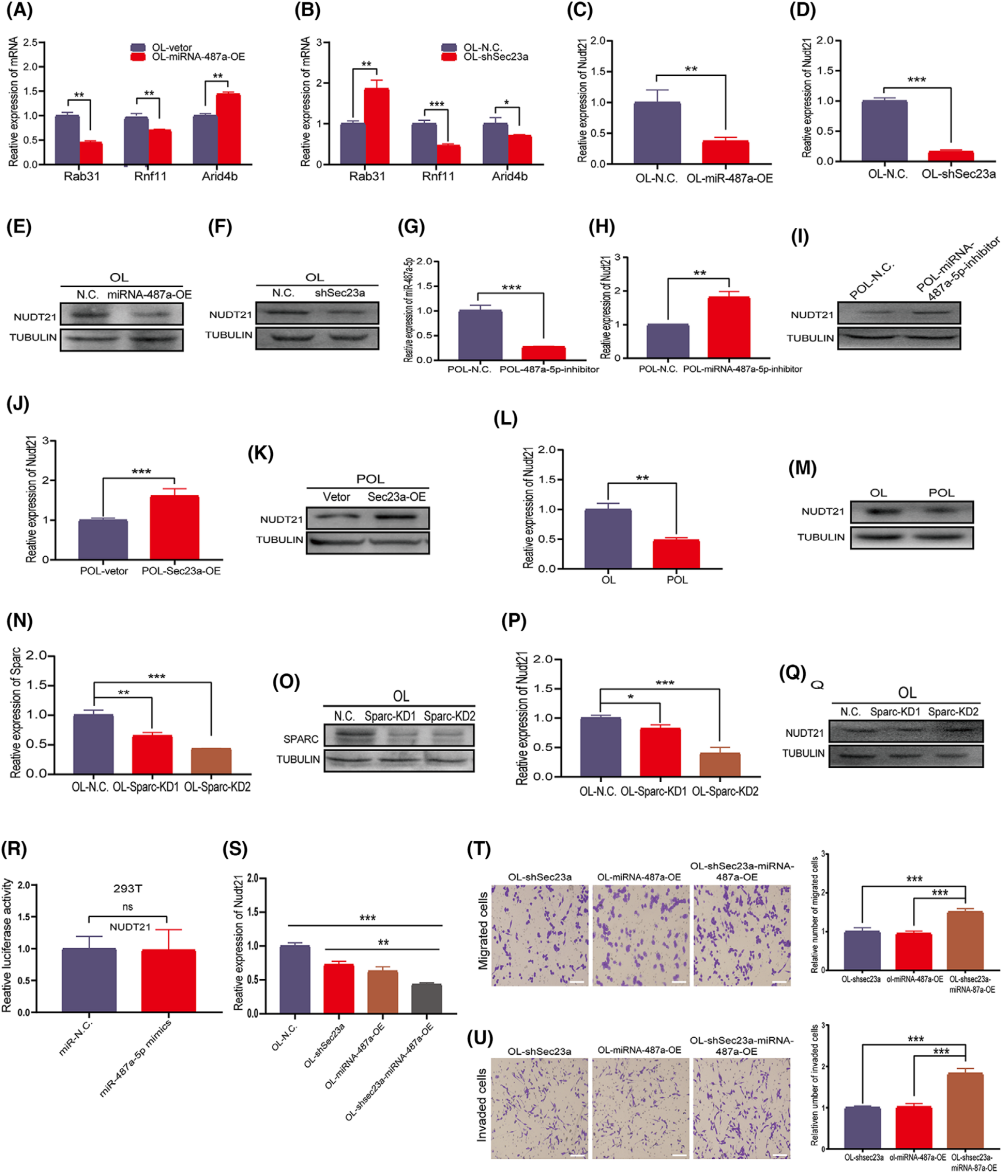
SEC23A and miR‐487a‐5p synergistically inhibit NUDT21 and promote OL cells to undergo polymetastasis. (A, B) The expression levels of Rnf11, Rab31, and Arid4b were detected in OL cells and OL‐miR‐487a‐OE cells (A) and in OL‐shSec23a cells (B); *n* = 3. (C, E) Nudt21 (C) mRNA expression and (E) protein expression in OL‐N.C. and OL‐miR‐487a‐OE cells; *n* = 3. (D, F) Nudt21 (D) mRNA expression and (F) protein expression in OL‐N.C. and OL‐shSec23a cells; *n* = 3. (G) Confirmation of inhibition efficiency of miR‐487a‐5p inhibitor treatment on miR‐487a‐5p expression in POL cells; *n* = 3. (H, I) Nudt21 (H) mRNA expression and (I) protein expression in POL‐N.C. and POL‐miR‐487a‐5p‐inhibitor cells; *n* = 3. (J, K) Nudt21 (J) mRNA expression and (K) protein expression in POL‐Vetor and POL‐Sec23a‐OE cells. (L, M) Nudt21 expression in OL and POL cells; (L) mRNA by qRT‐PCR; (M) protein by WB; *n* = 3. (N, O) Validation of Sparc knockdown. (N) mRNA by qRT‐PCR; (O) protein by WB; *n* = 3. (P, Q) Nudt21 expression in OL and OL‐si‐Sparc cells. (P) mRNA by qRT‐PCR; (Q) protein by WB; *n* = 3. (R) Assessment of miRNA‐487a‐5p and Nudt21 gene binding by the luciferase assay; *n* = 3. (S) Expression of Nudt21 in OL, OL‐shSec23a, OL‐miRNA‐487a‐OE, and OL‐shSec23a‐miRNA‐487a‐OE cells by PCR; *n* = 3. (T, U) OL‐shSec23a‐miR‐487a‐OE cells exhibited stronger (T) migration and (U) invasion abilities compared with that of OL‐shSec23a and OL‐miRNA‐487a‐OE cells (bar = 60 μm); *n* = 3. NC, negative control; OE, overexpression; OL, oligometastatic cell line; POL, polymetastatic cell line. *n* = 3: three times a particular experiment was replicated. Error bars indicated SD. *P < 0.05, **P < 0.01, ***P < 0.001 by *t*‐test.

### 3.5 Sec23a and miRNA‐487a‐5p synergistically inhibit Nudt21 to promote OL cells undergo polymetastasis

We first evaluated the relationship between the expression of Nudt21 and that of miR‐487a‐5p or Sec23a. We measured Nudt21 protein expression in OL cells that overexpressed miR‐487a‐5p (OL‐miR‐487a‐OE), or Sec23a was silenced (OL‐shSec23a). In both cases, Nudt21 expression was reduced compared with vector control OL cells (Fig. 5E,F). By contrast, in POL cells that either Sec23a was overexpressed (POL‐Sec23a‐OE) or miR‐487a‐5p was inhibited (POL‐miR‐487a‐inhibitor), Nudt21 mRNA and protein expression were significantly increased (Fig. 5G–K), respectively. Therefore, there is an inverse relationship between Nudt21 expression and Sec23a or miR‐487a‐5p expression. We also compared Nudt21 content in POL and OL cells that differ in metastatic colonization efficiency and we found that NUDT21 expression was significantly lower in POL cells (Fig. 5L,M), consistent with the reported tumor‐suppressor function of Nudt21 [25, 28].

So far, we showed that Sparc (Fig. 3) and Nudt21 expression (Fig. 5) are both Sec23a‐dependent. We next determined whether Nudt21 expression is regulated by the ‘Sec23a‐Sparc axis’. We assayed Nudt21 expression in Sparc‐knockdown OL cells (OL‐si‐Sparc) and found that Nudt21 mRNA expression is regulated by Sparc (Fig. 5N–P). However, no significant differences were observed in NUDT21 protein expression between OL‐si‐Sparc and OL cells (Fig. 5Q). This set of observations suggests that there might be a compensation mechanism at the translation level for NUDT21 protein expression upon Sparc knockdown. Since Sec23a regulates Nudt21 expression at both transcriptional and translational levels (Fig. 5) and Sparc only regulates Nudt21 mRNA expression, the possibility of Sec23a‐Sparc axis regulation of NUDT21 protein expression was excluded. While Nudt21 expression is regulated by Sec23a, the mechanisms involved are beyond the scope of this study and merit future investigation. To determine whether Nudt21 is a direct gene target of miR‐487a‐5p, we performed luciferase experiment. Luciferase experiment did not support Nudt21 to be a direct target of miRNA‐487a‐5p (Fig. 5R) but as an indirect and functional downstream effector.

Next, we constructed a set of OL‐derivative cell lines including OL‐shSec23a, OL‐miRNA‐487a‐OE, and OL‐shSec23a‐miRNA‐487a‐OE by lentivirus transfection. We used this set of genetically engineered cell lines to explore Nudt21‐mediated synergistic effects of Sec23a and miRNA‐487a‐5p on the metastatic competency of OL cells. Simultaneously knockdown of Sec23a and overexpression of miRNA‐487a‐5p in OL cells (OL‐shSec23a‐miRNA‐487a‐OE) resulted in more effective inhibition of Nudt21 expression (Fig. 5S) and consequently, more pronounced enhancement of *in vitro* invasiveness of OL cells than each alone (Fig. 5T,U).

**Fig. 6:**
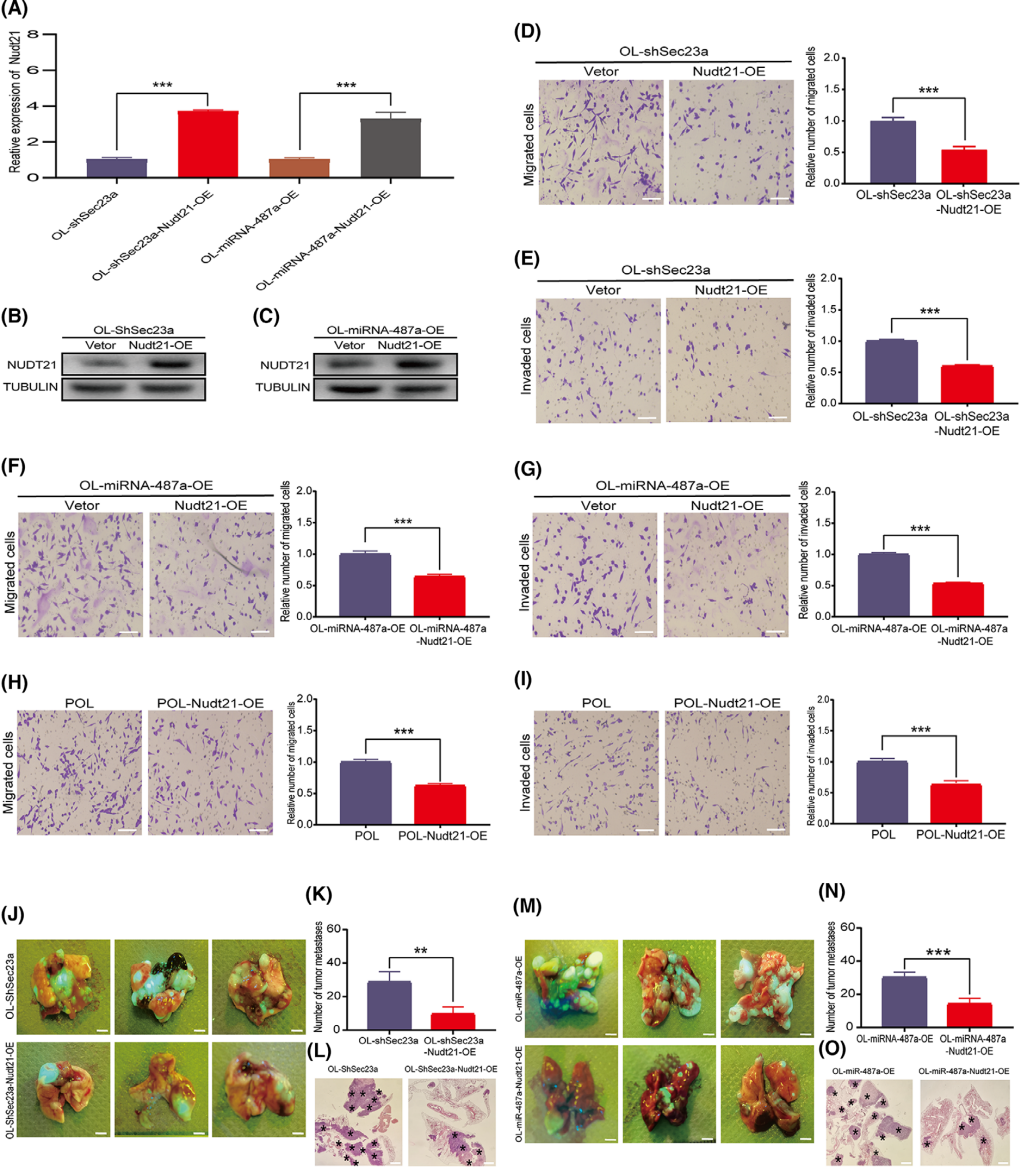
Nudt21 overexpression inhibits the metastatic competency of melanoma cells. (A–C) Validation of Nudt21 overexpression in OL‐shSec23a and OL‐miRNA‐487a‐OE cells, respectively. (A) PCR; (B, C) WB; *n* = 3. (D, E) Nudt21 overexpression inhibited the (D) migration and (E) invasion abilities of OL‐shSec23a cells (bar = 60 μm); *n* = 3. (F, G) Nudt21 overexpression inhibited the (F) migration and (G) invasion abilities of OL‐miRNA‐487a‐OE cells (bar = 60 μm); *n* = 3. (H, I) Nudt21 overexpression inhibited the (H) migration and (I) invasion abilities of POL cells (bar = 60 μm); *n* = 3. (J, M) Photographic representation of macroscopic metastases of NOD/SCID mice 4 weeks after tail vein injection of control cells and OL cells overexpressing Nudt21 cells (OL‐shSec23a, OL‐miRNA‐487a‐OE, OL‐shSec23a‐Nudt21‐OE, and OL‐miRNA‐487a‐Nudt21‐OE, bar = 3 mm); 5 mice per group. (K, N) Quantitative analysis of the superficial lung metastatic foci in mice injected with control OL cells or OL cells overexpressing Nudt21. (L, O) Visualization of intrapulmonary metastases in mice injected with control OL cells or OL cells overexpressing Nudt21 by H&E staining; Lung metastatic foci marked by* (bar = 2 mm). OE, overexpression; OL, oligometastatic cell line. *n* = 3: three times a particular experiment was replicated. Error bars indicated SD. **P < 0.01, ***P < 0.001 by *t*‐test.

To confirm that Nudt21 is tumor suppressive and mediates the pro‐metastatic activity of miR‐847a‐5p and the antimetastatic activity of Sec23a, OL‐shSec23a‐Nudt21‐OE, and OL‐miRNA‐487a‐Nudt21‐OE cell lines were constructed by lentivirus transfection (Fig. 6A–C) for conducting the rescue experiments both *in vitro* and *in vivo*. Compared with OL‐shSec23a and OL‐miRNA‐487a‐OE, Nudt21 overexpression effectively prevented the enhancement of *in vitro* invasiveness in OL‐shSec23a and OL‐miRNA‐487a‐OE cells, respectively (Fig. 6D–G). Similarly, compared with the highly metastatic POL cells in which Sec23a and Nudt21 expression was lower than that of OL cells and miR‐487a‐5p expression was higher, Nudt21 overexpression (POL‐Nudt21‐OE) significantly inhibited the *in vitro* invasiveness of POL cells (Fig. 6H,I). *In vivo* experiments also showed that mice injected with OL‐shSec23a‐Nudt21‐OE cells had significantly reduced lung metastases (Fig. 6J). Quantification of the number of surface metastatic nodules also confirmed that stable overexpression of Nudt21 effectively prevented Sec23a knockdown (OL‐shSec23a)‐induced oligometastatic to polymetastatic progression (Fig. 6K). Lung metastases in the Nudt21 overexpressed group were significantly less than those in the control group (Fig. 6L). By contrast, mice injected with OL‐miRNA‐487a‐Nudt21‐OE cells showed a significant reduction of metastases on the lung surface and intrapulmonary space (Fig. 6M–O).

Collectively, these results show that Nudt21 mediates the synergistic pro‐metastatic effect of POL‐exo and POL‐secretory proteins.

### 3.6 Nudt21 affects melanoma metastasis through glycolysis

The abnormal glycolysis of tumor cells is a hallmark of cancer state and is involved in tumor progression and metastasis [29, 30, 31]. We employed ‘dry‐lab discovery and wet‐lab validation’ approach to investigate whether highly metastatic POL cells can augment the metastatic competency of OL cells by modifying glycolysis within OL cells.

**Fig. 7:**
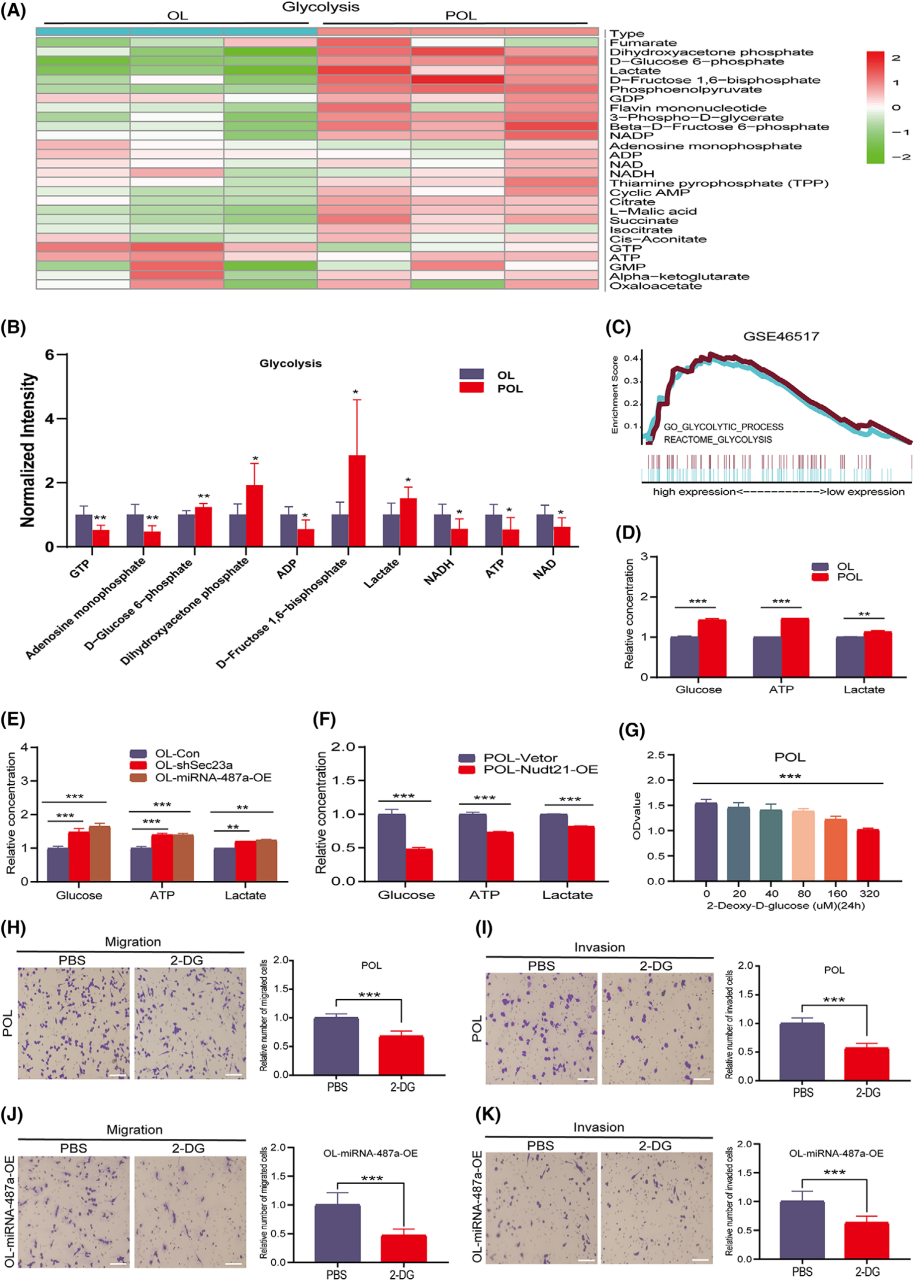
NUDT21 affects melanoma metastatic competency through glycolytic pathway. (A, B) Mass spectrometry analysis of glycolytic intermediates in POL and OL cells. (A) Heat map of glycolytic intermediates; (B) Histogram of key metabolites differences in the glycolytic pathway; *n* = 3. (C) GSEA analysis found that glycolysis‐related genes were overexpressed in the metastatic foci by GEO‐GSE46517 database. (D) Determination of glucose, ATP, and lactate content in OL and POL cells; *n* = 3. (E) Comparison of glucose, ATP, and lactate levels in OL, OL‐miRNA‐487a‐OE, and OL‐shSec23a cells; *n* = 3. (F) Nudt21 overexpression inhibited the level of glucose, ATP, and lactate in POL cells; *n* = 3. (G) Cytotoxicity analysis of glycolysis inhibitor 2‐Deoxy‐D‐glucose (2‐DG); *n* = 3. (H, I) 2‐DG (40 μm) inhibited the (H) migration and (I) invasion abilities of POL cells (bar = 60 μm); *n* = 3. (J, K) 2‐DG (40 μm) inhibited the (J) migration and (K) invasion abilities of OL‐miRNA‐487a‐OE cells (bar = 60 μm); *n* = 3. GEO, Gene Expression Omnibus; GSEA, gene set enrichment analysis; OE, overexpression; OL, oligometastatic cell line; POL, polymetastatic cell line. *n* = 3: three times a particular experiment was replicated. Error bars indicated SD. *P < 0.05, **P < 0.01, ***P < 0.001 by *t*‐test.

First, we used GEO‐GSE46517 melanoma dataset for GSEA enrichment analysis and found that glycolysis‐related genes were highly expressed in the metastatic melanoma lesions (Fig. 7C). Among the 106 glycolysis‐related genes extracted from MSigDB database, 27 genes were differentially expressed in the nonmetastatic and metastatic lesions of GEO‐GSE46517 (Fig. S4A,B).

Second, we performed clinical evaluation of the prognostic significance of the ‘glycolysis‐27‐gene set’ using the TCGA‐SKCM dataset. We found that 10 genes (PPP2R1A, STAT3, ADPGK, OGT, EIF6, PRKACB, MLXIPL, PRKACA, HIF1A, and GALK1) in the ‘glycolysis‐27‐gene set’ were prognostic markers for melanoma (Fig. S4C), of which 6 genes conferred favorable prognosis (ADPGK, HIF1A, STAT3, OGT, MLXIPL, and PRKACB; Fig. S4C, red) thus were tumor suppressive, and 4 genes predicted poorer prognosis (PRKACA, PPP2R1A, EIF6, GALK1; Fig. S4D, blue) thus were oncogenic. We named this prioritized gene set as ‘glycolysis‐prognosis‐10‐gene set’.

Third, we analyzed the correlation of the ‘glycolysis‐prognosis‐10‐gene set’ with Nudt21 using TCGA database. We found that Nudt21 was negatively correlated with 4 oncogenic genes (Fig. S4D, blue), and positively correlated with 5 tumor‐suppressive genes (Fig. S4D, red) in the ‘glycolysis‐prognosis‐10‐gene set’, respectively. This analysis was consistent with the tumor‐suppressor attributes of Nudt21 gene that were reported [25, 28] and we characterized above (Figs 5 and 6).

To summarize, following the workflow of miRNA databases analysis (reducing to 67 genes from a total of 3038 predicted target genes), LASSO machine learning and clinical evaluation (reducing to 4 genes from 67 predicted target genes), 4 common target genes of Sec23a and miRNA‐487a‐5p genes (RNF11, NUDT21, RAB31, and ARID4B) in the Lasso ‘risk prediction model’ were prioritized. Expression validation and literature evaluation have finally identified NUDT21 for mechanistic investigation. Collectively, through dry‐lab analyses, we have pinned the tumor‐suppressor function of Nudt21 to the hallmark of human cancer—deregulated glycolysis. We have also identified the ‘glycolysis‐prognosis‐10‐gene set’ that has prognostic significance for melanoma for future clinical validation and translation.

Next, we used the experimental system to validate the dry‐lab finding that POL cells utilize Nudt21 to effectively enhance the metastatic competency of OL cells via the ‘secretory protein‐Sec23a‐Nudt21 axis’ and the ‘exosomal miRNA‐487a‐5p‐Nudt21 axis’ by regulating glycolysis in OL.

First, we analyzed glycolysis activity in POL and OL cells by measuring the glycolytic intermediates using mass spectrometry (Section 2). Glycolytic activity was significantly increased in POL cells compared with that in OL cells. This was evident by the significant increase in the level of the key metabolites of glycolysis, such as D‐glucose 6‐phosphate, dihydroxyacetone phosphate, D‐fructose 1,6‐bisphosphate in POL cells (Fig. 7A,B).

Second, we measured the glycolysis intermediates (glucose, ATP, and lactate) in POL and OL cells using the glycolysis kit (Section 2). Consistent with mass spectrometry analysis, the content of glucose, ATP, and lactate was higher in POL cells than that in OL cells, respectively (Fig. 7D). These results indicate that the difference in glycolysis is a new feature of metastatic cells that have different metastatic colonization capability. These findings also prompted us to hypothesize that ‘glycolysis promotes oligo‐ to polymetastatic progression’.

Third, to elucidate the role of glycolysis in oligometastatic to polymetastatic progression, we measured the contents of key glycolysis intermediates in OL, OL‐shSec23a, and OL‐miRNA‐487a‐OE cells using the glycolysis kit. Compared with OL cells, the contents of glucose, ATP, and lactate were significantly higher in OL‐miRNA‐487a‐OE and OL‐shSec23a cells (Fig. 7E). Meanwhile, compared with POL cells, overexpression of Nudt21 in POL cells (POL‐Nudt21‐OE) significantly reduced the glycolytic activity in POL cells (Fig. 7F). These findings indicate that glycolysis in OL and POL cells is Nudt21‐dependent. Treatment of POL cells and OL‐miRNA‐487a‐OE cells with 40 μm glycolysis inhibition 2‐deoxy‐D‐glucose (2‐DG) for 24 h resulted in significantly reduced *in vitro* invasiveness (Fig. 7G–K). Therefore, glycolysis augments the metastatic capability of metastatic melanoma cells.

### 3.7 The additional wet‐lab validation in human melanoma cell line

We have demonstrated that polymetastatic POL cells can utilize the miRNA‐487a‐5p to augment the polymetastatic competency of the OL cells, which downregulated Nudt21 and promote the glycolytic pathway. Considering the rigor of experimental conclusions and the clinical practice, we further choose the human melanoma cell line A375 to perform additional wet‐lab validation. We detected the expression of NUDT21 in A375‐miRNA‐487a‐5p‐mimics and A375‐miRNA‐487a‐5p‐inhibitor cell samples, and the results showed that NUDT21 was downregulated by miRNA‐487a‐5p in A375 (Fig. S5A,B). Second, the invasiveness of A375 cells was significantly increased upon miRNA‐487a‐5p‐mimics treatment compared with the control, while A375‐miRNA‐487a‐5p‐inhibitor cells showed weakened metastatic abilities (Fig. S5C,D). Further, based on the preverified conclusion that the contents of glucose, ATP, and lactate were significantly higher in OL‐miRNA‐487a‐OE compared with OL cells, we speculate that this conclusion also holds in A375 cells. The contents of glucose, ATP, and lactate were also detected in A375‐NC, A375‐miRNA‐487a‐5p‐mimics, and A375‐miRNA‐487a‐5p‐inhibitor cell samples, of which the results confirmed our predictions (Fig. S5E–G). And the treatment of A375‐miRNA‐487a‐5p‐mimics cells with 40 μm glycolysis inhibition 2‐deoxy‐D‐glucose (2‐DG) for 24 h was found to result in significantly reduced *in vitro* invasiveness (Fig. S5H,I). Thus, we confirmed the key findings in human melanoma cell line A375.

### 3.8 POL‐secreted S100A11 inhibits Sec23a in OL cells

**Fig. 8:**
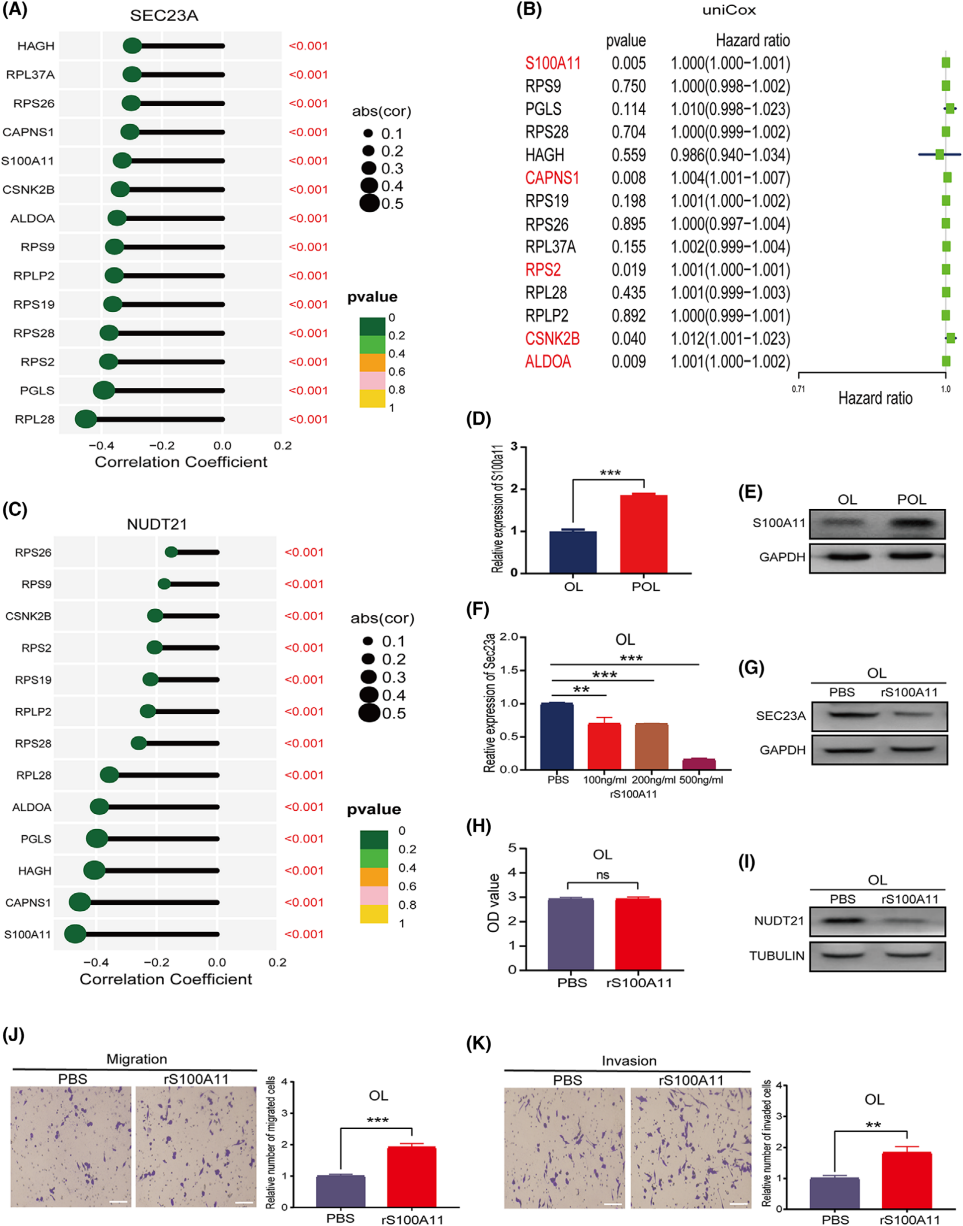
S100A11 in the POL‐derived secretome is an upstream secretory protein that regulates Sec23a expression in OL cells. (A) Correlation analysis of Sec23a with 14 genes of the secreted proteins. (B) Univariate prognostic analysis of 14 secreted proteins in the TCGA melanoma dataset. (C) Correlation analysis between Nudt21 and 14 secreted protein genes in TCGA‐SKCM database. (D, E) S100A11 expression in OL and POL cells; (D) mRNA by qRT‐PCR; (E) protein by WB; *n* = 3. (F, G) rS100A11 attenuates expression of Sec23a gene in OL cells; (F) mRNA by qRT‐PCR; (G) protein by WB; *n* = 3. (H) Cytotoxicity test of recombinant protein S100A11 (rS100A11); *n* = 3. (I) rS100A11 inhibits expression of NUDT21 in OL cells; *n* = 3. (J, K) Treatment with rS100A11 protein (500 g·mL^−1^) inhibited the (J) migration and (K) invasion abilities of OL cells (bar = 60 μm); *n* = 3. OL, oligometastatic cell line; POL, polymetastatic cell line; TCGA, The Cancer Genome Atlas. *n* = 3: three times a particular experiment was replicated. Error bars indicated SD. **P < 0.01, ***P < 0.001 by *t*‐test.

So far, we have shown that POL cells can transfer their metastatic competency to OL cells through synergistic targeting of Nudt21 via the secretory protein pathway and exosomal transfer of miR‐487a‐5p. However, we have yet to identify among 198 secretory proteins detected in POL condition medium [14] that inhibits Sec23a expression in OL. We achieved this goal by using the ‘Dry‐lab discovery and wet‐lab validation’ approach:

First, Correlation analysis between the genes of the 198 secretory proteins and Sec23a using TCGA‐SKCM dataset found that Sec23a mRNA expression was significantly and negatively correlated with the expression of 14‐secretory proteins (S100A11, RPS9, PGLS, RPS28, HAGH, CAPNS1, RPS19, RPS26, RPL37A, RPS2, RPL28, RPLP2, CSNK2B, ALDOA) (*R* < −0.3, *P* < 0.001) (Fig. 8A). There was no overlap between the ‘14‐secretory protein gene list’ and the ‘Sec23‐regulated secretory proteins list’ based on protein mass spectrum profiling of OL and OL‐shSec23a cells culture medium. This result indicates that the secretion of the ‘14‐secretory proteins’ is not regulated by Sec23a (Fig. S3B).

Second, clinical relevance analysis prioritized 5 of the 14 genes (S100A11, CAPNS1, RPS2, CSNK2B, and ALDOA) that conferred poor prognosis in TCGA‐SKCM patients (Fig. 8B).

Third, we performed a correlation analysis of the ‘14‐secretory protein gene list’ and Nudt21—the downstream mediator of Sec23a. Nudt21 was negatively correlated with 13 genes of the ‘14‐secretory protein gene list’, among which S100A11 had the strongest negative correlation (*R* < −0.4, *P* < 0.001) (Fig. 8C). *In this way, we prioritized S100A11 from 198 secretory proteins of POL and made the prediction that POL‐secreted S100A11 inhibits Sec23a in OL cells*.

Fourth, we conducted a set of experiments to validate this prediction: (a) The mRNA and protein expression of S100A11 was significantly higher in POL cells than that in OL cells (Fig. 8D,E). (b) Treatment of OL cells with recombinant S100A11 (rS100A11) lead to a dose‐dependent inhibition of Sec23a mRNA and protein expression (Fig. 8F,G). (c) NUDT21 protein expression was significantly decreased in OL cells upon treatment with 500 ng·mL^−1^ rS100A11 (Fig. 8H,I). (d) *In vitro* invasiveness of OL cells was significantly enhanced upon treatment with 500 ng·mL^−1^ rS100A11 (Fig. 8J,K). These findings collectively confirmed that S100A11 can inhibit Sec23a expression and augment the invasiveness of OL cells. Therefore, POL cells can transfer their polymetastatic competency to the OL cells via the ‘S100A11‐Sec23a‐Nudt21’ secretory protein mechanism.

In summary, we have revealed for the first time that polymetastatic POL cells can utilize both the secretory protein pathway (S100A11‐Sec23a) and the exosomal crosstalk (miR‐487a‐5p) synergistically to augment the polymetastatic competency of the OL cells. This is achieved via co‐targeting the tumor‐suppressor activity of Nudt21 in OL cells thus synergistically relieving the inhibition of Nudt21 on OL metastasis. We thus have characterized a new molecular mechanism underlying oligometastatic to polymetastatic progression. In addition, we have identified deregulated glycolysis as one of the metabolic changes that may regulate metastatic colonization efficiency. The ‘glycolysis‐prognosis‐10‐gene set’ that has independent prognostic significance for melanoma was identified and merits future clinical validation.

## 4 Discussion

**Fig. 9:**
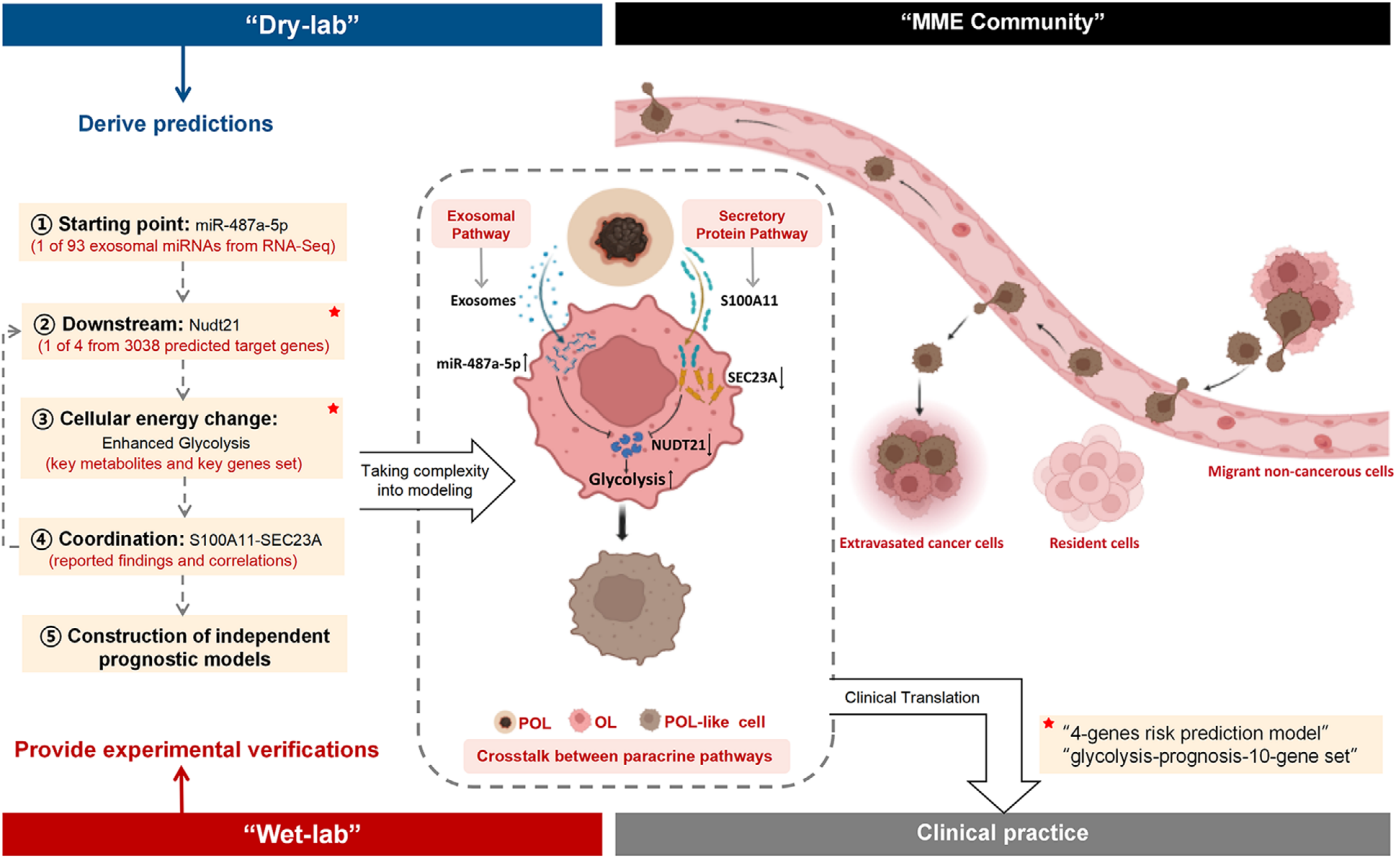
Schematic summary of the ‘dry‐lab discovery/wet‐lab validation’ approach and key findings. The story of cancer metastasis is complex in the time of making, in the space of transformation, and most importantly, in the interactions between and among all who are involved in such a making. At the distant organ, metastatic microenvironment (MME) is a snapshot of a ‘community’ in which noncancerous cells (resident cells of the distant organ and migrant noncancerous cells—vascular endothelial cells, fibroblasts, various types of immune cells) have been enlisted and trained by the invading cancer cells to co‐operate with the goal of succeeding in metastatic colonization, that is, the establishment of clinically meaningful metastatic lesions. In addition to the dynamic changes in MME, successful metastasis requires material transmission through paracrine pathways between cells of the same type or the different. To embrace the complexity of metastasis, the ‘dry‐lab discovery/wet‐lab validation’ approach was applied to study the crosstalk between the two paracrine pathways—the secretory protein pathway and the exosomal pathway in facilitating the interactions between oligo‐ (OL) and polymetastatic (POL) melanoma cancer cells that differ in metastatic competency. POL cells can utilize both the secretory protein pathway (S100A11‐Sec23a) and the exosomal pathway (miR‐487a‐5p) synergistically to transfer their polymetastatic competency to OL cells, which switches the metastatic phenotype of OL from oligo‐ to polymetastasis. This is achieved via ‘S100A11‐Sec23a’ and miR‐487a‐5p co‐targeting the tumor‐suppressor activity of Nudt21 in OL cells thus synergistically relieving the inhibition of Nudt21 on glycolysis. Identification of two gene sets (4‐genes risk prediction model and glycolysis‐prognosis 10 gene set) that each conferring independent prognosis in melanoma has demonstrated the capability of the ‘dry‐lab discovery/wet‐lab validation’ approach in accelerating the process from knowledge discovery to clinical translation by taking into consideration of the complexity of cancer and clinical relevance.

In the present study, we show that the approach is efficient in studying the crosstalk between the two paracrine pathways (the secretory pathway and the exosomal pathway) in the context of facilitating the communications between melanoma cancer cells with different metastatic competency during metastatic colonization. We have made the following mechanistic predictions otherwise will not be possible (Fig. 9):

Firstly, we prioritized miR‐487a‐5p from the 93 miRNAs that exhibited higher expression in POL‐exo for mechanistic investigation. Although the oncogenic activity of miR‐487a‐5p in hepatocellular carcinoma has been reported [32, 33], the function of miR‐487a‐5p in the context of exosomal transfer and in melanoma is unknown prior to this study.

Secondly, we prioritized Nudt21 from the 3038 predicted gene targets of miR‐487a‐5p as the common mediator of the synergistic interaction between the exosomal (miRNA‐487a‐5p) and secretory protein (S100A11‐Sec23a) pathways. This allows us to demonstrate for the first time that melanoma cancer cells with different metastatic competency can interact by maximizing the two paracrine pathways. LASSO machine learning was used to fully consider the relationships between target genes and prognostic features. The tumor‐suppressive function of Nudt21 that we predicted and validated is consistent with the literature [25, 26, 27, 28].

Thirdly, we predicted and validated that Nudt21 affects melanoma metastasis through glycolysis. Through dry‐lab analyses, we have pinned the tumor‐suppressor function of Nudt21 to the deregulated glycolysis. This prediction was validated with wet‐lab experimentations.

Fourthly, we prioritized S100A11 from 198 secretory proteins of POL and made the prediction that POL‐secreted S100A11 inhibits Sec23a in OL cells. Our previous studies have shown that Sec23a inhibits metastatic colonization by regulation of the secretome [14, 24]. Thus, Sec23a is the initiator of the tumor‐suppressive activity of the Sec23a‐dependent secretome. Here, we show that Sec23a is the effector within recipient cells (OL cells). Therefore, we speculate that within the proteins secreted by POL cells, there exists the negative regulator of Sec23a in OL cells. With literature evaluation [34, 35] and wet‐lab verification, we excluded the possibility of regulation of Sec23a by exosomes of the POL (POL‐exo). By contrast, we show that secretory proteins derived from POL cells can regulate the expression of Sec23a in OL cells. Combining the bioinformatics analyses and the secretory protein library of OL/POL cells established by our laboratory [16], we predicted and validated that S100A11 secreted by POL cells was the upstream regulator of Sec23a.

Fifthly, we identified ‘4‐genes risk prediction model’ (RNF11, NUDT21, RAB31, and ARID4B) and the ‘glycolysis‐prognosis‐10‐gene set’ that each confers *independent* prognostic significance for melanoma. The hazard ratios of these two gene sets are significantly higher than that of TNM. Thus, the dry‐lab discovery approach can generate new clinically meaningful knowledge that is ready for further clinical validation and translation.

Finally, the involvement of deregulated glycolysis in metastasis has long been appreciated [29, 36, 37, 38]. For example, the increase of tumor glycolysis and lactic acid production weakens the antitumor immunity mediated by T cells in melanoma microenvironment, thus promoting tumor progression [39, 40]. However, glycolysis is a complex metabolic process. It is beyond the reach of the wet‐lab investigation to examine the correlation of the gene of interest with all of the known glycolysis‐related genes and at the same time, taken into consideration of the clinical relevance. In this study, while comprehensive dry‐lab analyses have discovered and predicted the regulatory function of Nudt21 on glycolysis, wet‐lab experimentation has provided the proof by measuring changes in the few key metabolites of glycolysis when Nudt21 expression is altered. Thus, we show for the first time that an increase in glycolysis is the mechanism underlying the pro‐metastatic activity of POL cells on the OL cells via the crosstalk between the secretory protein and exosomal paracrine pathways. In addition, we have identified deregulated glycolysis as one of the metabolic changes that may regulate metastatic colonization efficiency. We believe that this approach can be utilized to investigate the involvement of other metabolic processes in mediating the communications between cancer cells and stromal cells, as well as between heterogeneous cancer cells in the MME.

## 5 Conclusion

In this study, the paired M14 melanoma derivative oligometastatic cells (OL) and polymetastatic cells (POL) that differ in metastatic colonization efficiency *in vivo* were used [16]. We have applied the ‘dry‐lab discovery/wet‐lab validation’ approach that we have developed to simulate the interactions between heterogeneous cancer cells (OL and POL) in the MME and to illustrate how can OL and POL cells exploit the secretory proteins and exosomal crosstalk to facilitate their optimal interactions. We have revealed for the first time that polymetastatic POL cells can utilize both the secretory protein pathway (S100A11‐Sec23a) and the exosomal pathway (miR‐487a‐5p) synergistically to transfer their polymetastatic competency to the low‐metastatic OL cells, which switches the metastatic phenotype of OL from oligo‐ to polymetastasis. This is achieved via ‘S100A11‐Sec23a’ and miR‐487a‐5p co‐targeting the tumor‐suppressor activity of Nudt21 in OL cells thus synergistically relieving the inhibition of Nudt21 on glycolysis. With the aid of the new research approach, we have characterized a new molecular mechanism underlying oligo‐ to polymetastatic progression. In addition, we have identified deregulated glycolysis as one of the metabolic changes that may regulate metastatic colonization efficiency. Further, we have identified two gene sets, each conferring independent prognosis in melanoma that has the potential for clinical translation.

## Conflict of interest

The authors declare no conflict of interest.

## Author contributions

BZ and YC performed the experiments and analyzed data. BZ and YC contributed to the writing of this manuscript. HC, QZ, ZS, DL, XL, and YZ participated in the conduction of this study. HRX and JW designed this study, oversaw the execution of this study, and contributed to the writing and revision of this manuscript.

## Supporting information


**Fig. S1.** Confirmation of different metastatic competency of the OL and POL cell models. (A, B) Comparison of the *in vitro* (A) migration and (B) invasion abilities of OL and POL cells (bar = 60 μm); n = 3. (C) Comparison of colony formation abilities of OL and POL cells; n = 3. (D) Comparison of the *in vitro* proliferative abilities of OL and POL cells; n = 3. (E) The expression of exosome markers ALIX, CD63, and CD81 of OL and POL cells was detected by western blot; n = 3. (F) Electron microscopy examination of exosomes from OL and POL cells; Left: OL exosomes; Right: POL exosomes; n = 3. (G) Particle size measurement of OL and POL exosomes by nanoparticle tracking analysis (NTA); n = 3. OL—oligometastatic cell line; POL—polymetastatic cell line. n = 3: three times a particular experiment was replicated. Error bars indicated SD. *p < 0.05, **p < 0.01, ***p < 0.001 by *t*‐test.

**Fig. S2.** RT‐qPCR validation of overexpressed miRNAs in OL and POL cells and their exosomes. (A) RT‐qPCR validation of 14 differentially expressed miRNAs in OL and POL cells; n = 3. (B) RT‐qPCR validation of miRNA‐488, miRNA‐487a‐5p, and miRNA‐411‐5p in OL and POL cells and their respective exosomes; n = 3. (C) Photographic representation of macroscopic lung metastases of NOD/SCID mice 3 weeks after tail vein injection of control cells and miRNA‐487a overexpressed cells (OL‐Vetor and OL‐miRNA‐487a‐OE, bar = 3 mm); 5 mice per group. OL—oligometastatic cell line; POL—polymetastatic cell line; OE—overexpression. n = 3: three times a particular experiment was replicated. Error bars indicated SD. *p < 0.05, **p < 0.01, ***p < 0.001 by *t*‐test.

**Fig. S3.** SPARC is a downstream gene regulated by SEC23A. (A) The target genes of miRNA‐487a‐5p were predicted using TargetScan, miRTarBase, and miRDB databases. (B) Quantitative analysis of differentially downregulated secreted proteins in OL‐N.C. and OL‐shsec23a media. (C) Gene correlation analysis between SPARC and SEC23A in TCGA‐SKCM (R > 0.4, P < 0.05). (D, F) mRNA expression of Sparc in OL, OL‐shSec23a, POL, and POL‐Sec23a‐OE cells; n = 3. (E, G) Protein level of SPARC in OL, OL‐shSec23a, POL, and POL‐Sec23a‐OE cells; n = 3. (H, I) Sparc knockdown enhanced the (H) migration and (I) invasion abilities of OL cells (bar = 60 μm); n = 3. (J, K) Treatment with rSPARC protein (8ug/ml) inhibited the (J) migration and (K) invasion abilities of OL cells (bar = 60 μm); n = 3. OL—oligometastatic cell line; POL—polymetastatic cell line; NC—negative control. n = 3: three times a particular experiment was replicated. Error bars indicated SD. *p < 0.05, **p < 0.01, ***p < 0.001 by *t*‐test.

**Fig. S4.** Clinical prognostic evaluation of glycolysis‐related gene set in melanoma. (A, B) Differential expression analysis of 106 glycolysis‐related genes in the GEO‐GSE46517 dataset. (A) Heat map; (B) volcano map. (C) Univariate prognostic analysis of 27 differentially expressed glycolytic genes in the TCGA‐SKCM database. Red: positive correlation with prognosis, blue: negative correlation with prognosis. (D) Correlations of the 10 prognostic marker genes of the ‘glycolysis‐27‐gene set’ with Nudt21. GEO—Gene Expression Omnibus; TCGA—The Cancer Genome Atlas; GSEA—gene set enrichment analysis.

**Fig. S5.** NUDT21 affects melanoma metastatic competency through glycolytic pathway. (A, B) The expression of (A) miRNA‐487a‐5p and (B) NUDT21 in A375 cells transfected with miRNA‐487a‐5p‐mimics or inhibitor was detected by RT‐qPCR; n = 3. (C, D) miRNA‐487a‐5p‐mimic treatment significantly enhanced the (C) migration and (D) invasion abilities of A375 cells, while A375‐miRNA‐487a‐5p‐inhibitor cells showed weakened invasiveness (bar = 210 μm); n = 3. (E‐G) The contents of (E) glucose, (F) ATP, and (G) lactate were significantly higher in A375‐miRNA‐487a‐5p‐mimics cells, while reduced in A375‐miRNA‐487a‐5p‐inhibitor cells; n = 3. (G, H) 2‐DG (40 μM) inhibited the (G) migration and (H) invasion abilities of A375‐miRNA‐487a‐5p‐mimics cells (bar = 210 μm); n = 3. n = 3: three times a particular experiment was replicated. Error bars indicated SD. *p < 0.05, **p < 0.01, ***p < 0.001 by *t*‐test.

**Table S1.** The primer sequences.

## Data Availability

The datasets used and/or analyzed during the current study are available from the corresponding author upon reasonable request.
