# Supplementary figures and images for: Synergistic inhibition of NUDT21 by secretory S100A11 and exosomal miR‐487a‐5p promotes melanoma oligo‐ to poly‐metastatic progression
Source: Mol Oncol. 2023 Jul 1;17(12):2743–66. doi: 10.1002/1878-0261.13480 (PMC10701767; doi:10.1002/1878-0261.13480)

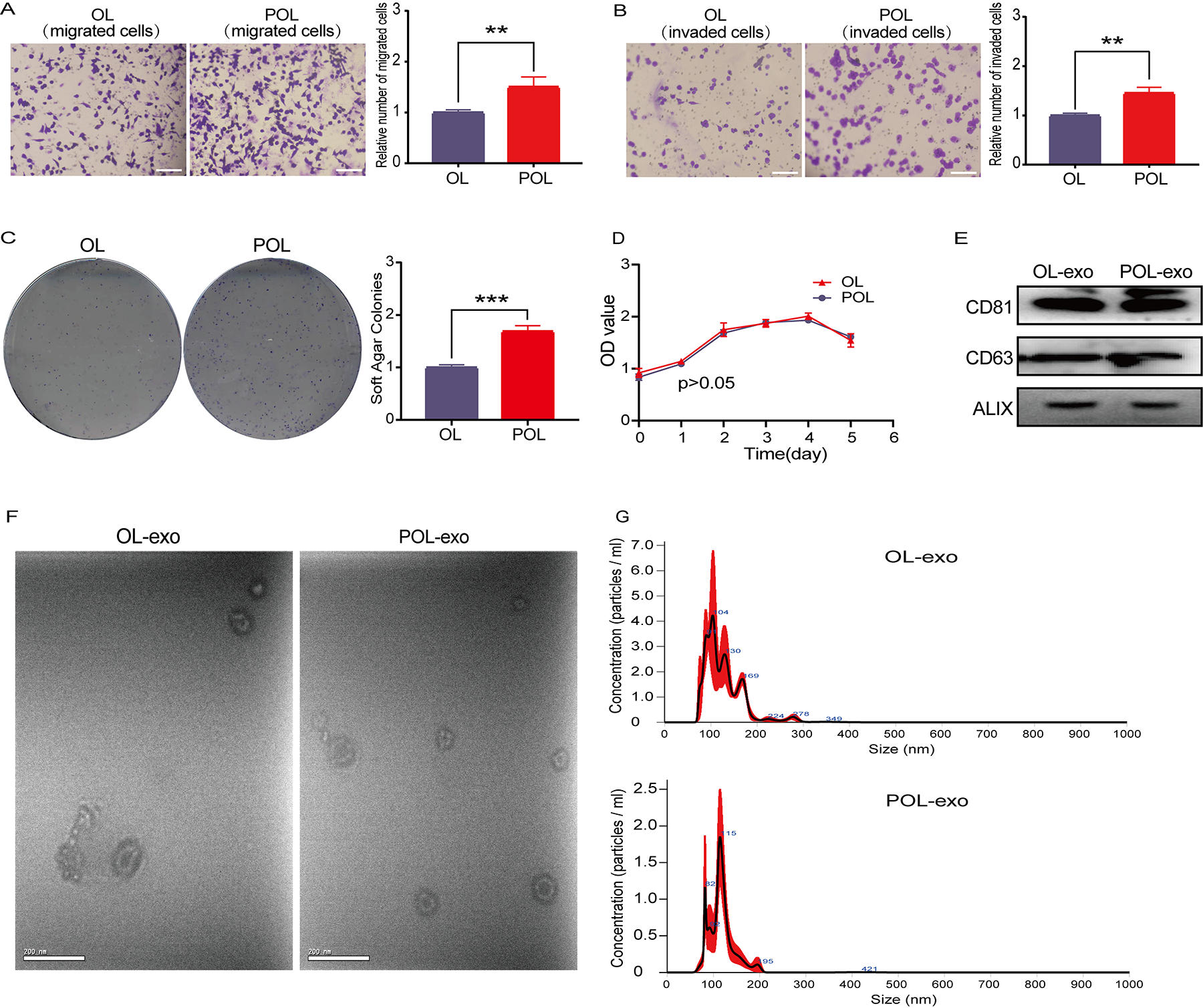

Supplement: Supplementary file 1 — Fig. S1. Confirmation of different metastatic competency of the OL and POL cell models. (A, B) Comparison of the in vitro (A) migration and (B) invasion abilities of OL and POL cells (bar = 60 μm); n = 3. (C) Comparison of colony formation abilities of OL and POL cells; n = 3. (D) Comparison of the in vitro proliferative abilities of OL and POL cells; n = 3. (E) The expression of exosome markers ALIX, CD63, and CD81 of OL and POL cells was detected by western blot; n = 3. (F) Electron microscopy examination of exosomes from OL and POL cells; Left: OL exosomes; Right: POL exosomes; n = 3. (G) Particle size measurement of OL and POL exosomes by nanoparticle tracking analysis (NTA); n = 3. OL—oligometastatic cell line; POL—polymetastatic cell line. n = 3: three times a particular experiment was replicated. Error bars indicated SD. *p < 0.05, **p < 0.01, ***p < 0.001 by t‐test. [file MOL2-17-2743-s001.tif]

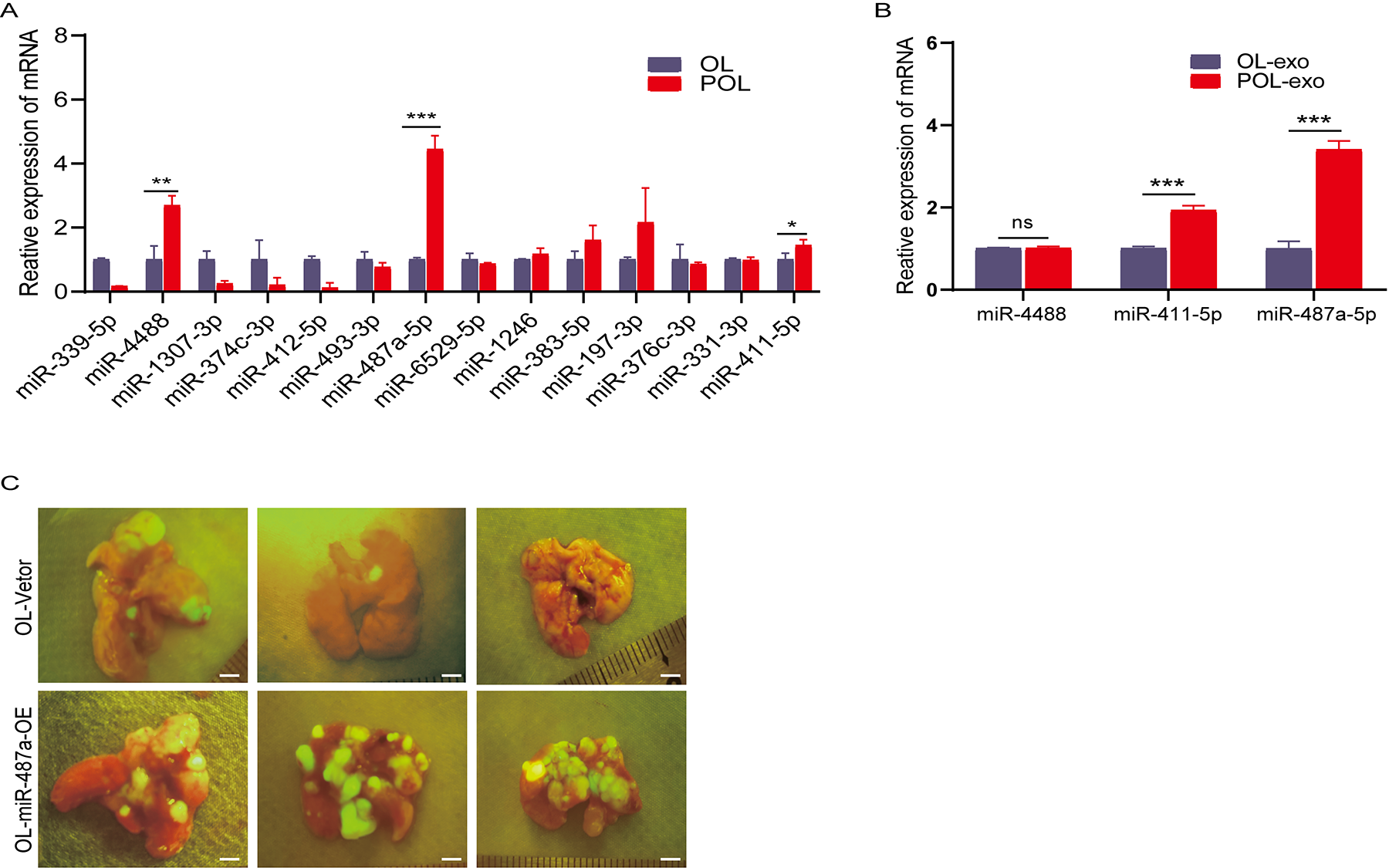

Supplement: Supplementary file 2 — Fig. S2. RT‐qPCR validation of overexpressed miRNAs in OL and POL cells and their exosomes. (A) RT‐qPCR validation of 14 differentially expressed miRNAs in OL and POL cells; n = 3. (B) RT‐qPCR validation of miRNA‐488, miRNA‐487a‐5p, and miRNA‐411‐5p in OL and POL cells and their respective exosomes; n = 3. (C) Photographic representation of macroscopic lung metastases of NOD/SCID mice 3 weeks after tail vein injection of control cells and miRNA‐487a overexpressed cells (OL‐Vetor and OL‐miRNA‐487a‐OE, bar = 3 mm); 5 mice per group. OL—oligometastatic cell line; POL—polymetastatic cell line; OE—overexpression. n = 3: three times a particular experiment was replicated. Error bars indicated SD. *p < 0.05, **p < 0.01, ***p < 0.001 by t‐test. [file MOL2-17-2743-s003.tif]

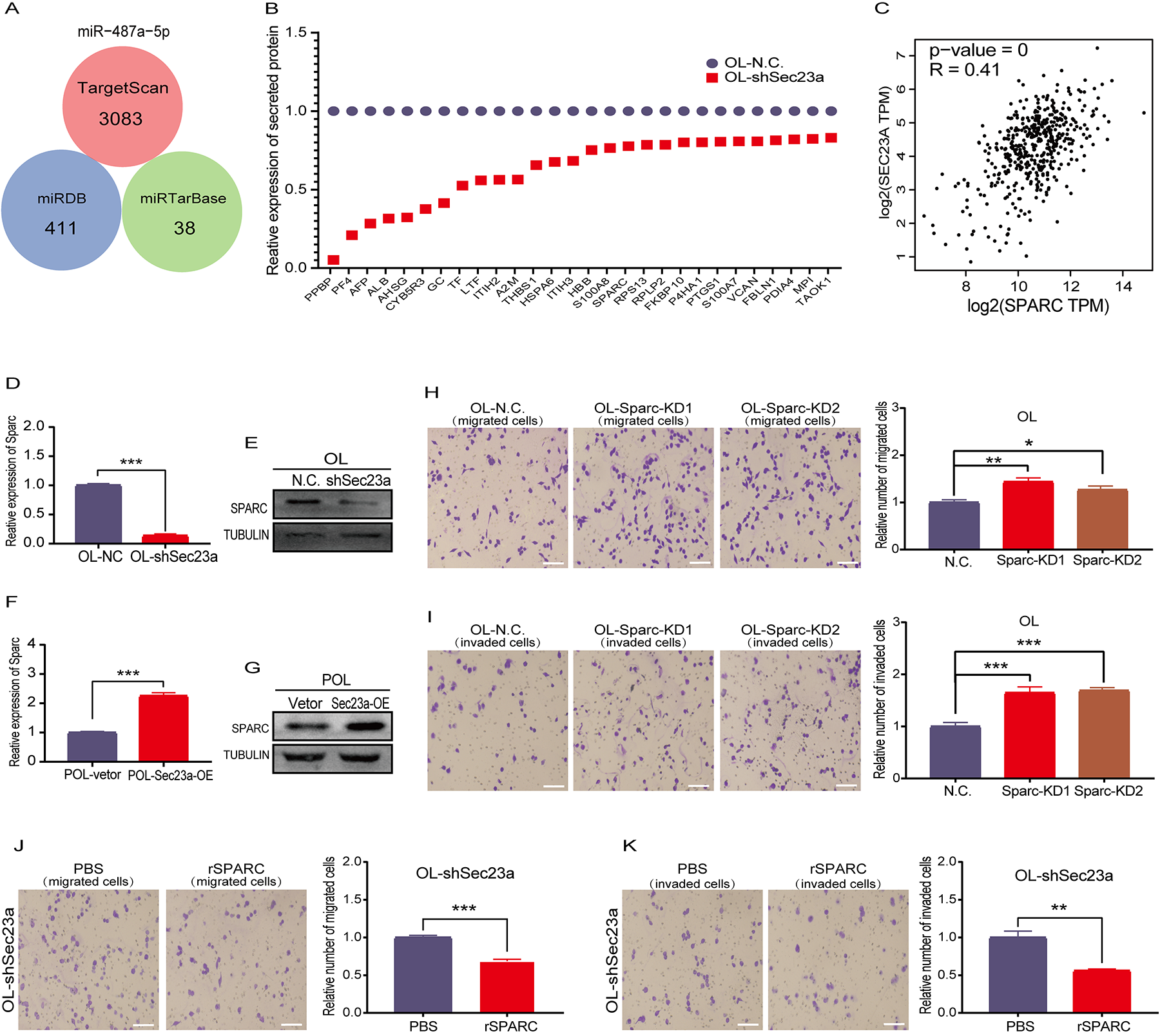

Supplement: Supplementary file 3 — Fig. S3. SPARC is a downstream gene regulated by SEC23A. (A) The target genes of miRNA‐487a‐5p were predicted using TargetScan, miRTarBase, and miRDB databases. (B) Quantitative analysis of differentially downregulated secreted proteins in OL‐N.C. and OL‐shsec23a media. (C) Gene correlation analysis between SPARC and SEC23A in TCGA‐SKCM (R > 0.4, P < 0.05). (D, F) mRNA expression of Sparc in OL, OL‐shSec23a, POL, and POL‐Sec23a‐OE cells; n = 3. (E, G) Protein level of SPARC in OL, OL‐shSec23a, POL, and POL‐Sec23a‐OE cells; n = 3. (H, I) Sparc knockdown enhanced the (H) migration and (I) invasion abilities of OL cells (bar = 60 μm); n = 3. (J, K) Treatment with rSPARC protein (8ug/ml) inhibited the (J) migration and (K) invasion abilities of OL cells (bar = 60 μm); n = 3. OL—oligometastatic cell line; POL—polymetastatic cell line; NC—negative control. n = 3: three times a particular experiment was replicated. Error bars indicated SD. *p < 0.05, **p < 0.01, ***p < 0.001 by t‐test. [file MOL2-17-2743-s002.tif]

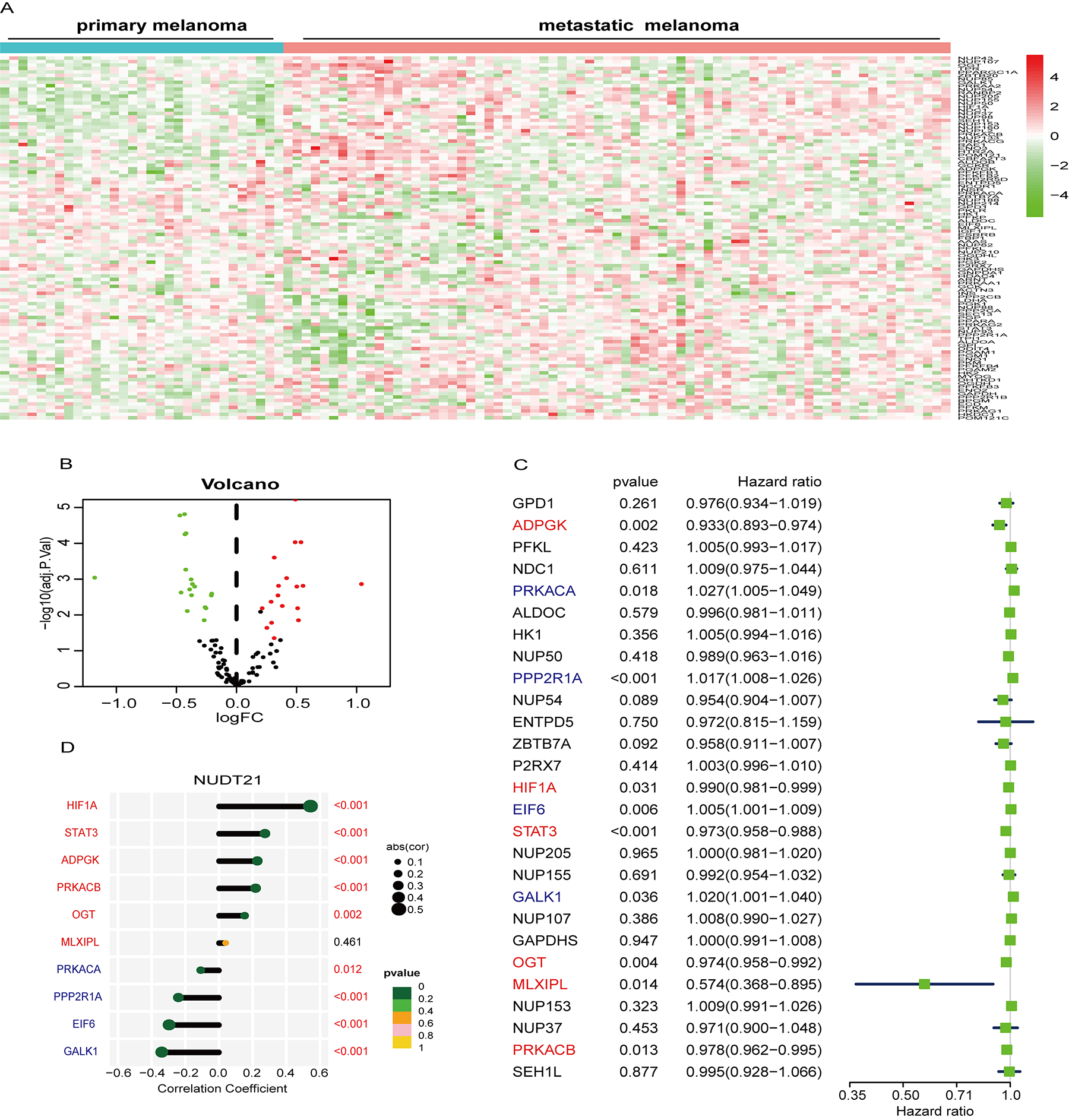

Supplement: Supplementary file 4 — Fig. S4. Clinical prognostic evaluation of glycolysis‐related gene set in melanoma. (A, B) Differential expression analysis of 106 glycolysis‐related genes in the GEO‐GSE46517 dataset. (A) Heat map; (B) volcano map. (C) Univariate prognostic analysis of 27 differentially expressed glycolytic genes in the TCGA‐SKCM database. Red: positive correlation with prognosis, blue: negative correlation with prognosis. (D) Correlations of the 10 prognostic marker genes of the ‘glycolysis‐27‐gene set’ with Nudt21. GEO—Gene Expression Omnibus; TCGA—The Cancer Genome Atlas; GSEA—gene set enrichment analysis. [file MOL2-17-2743-s005.tif]

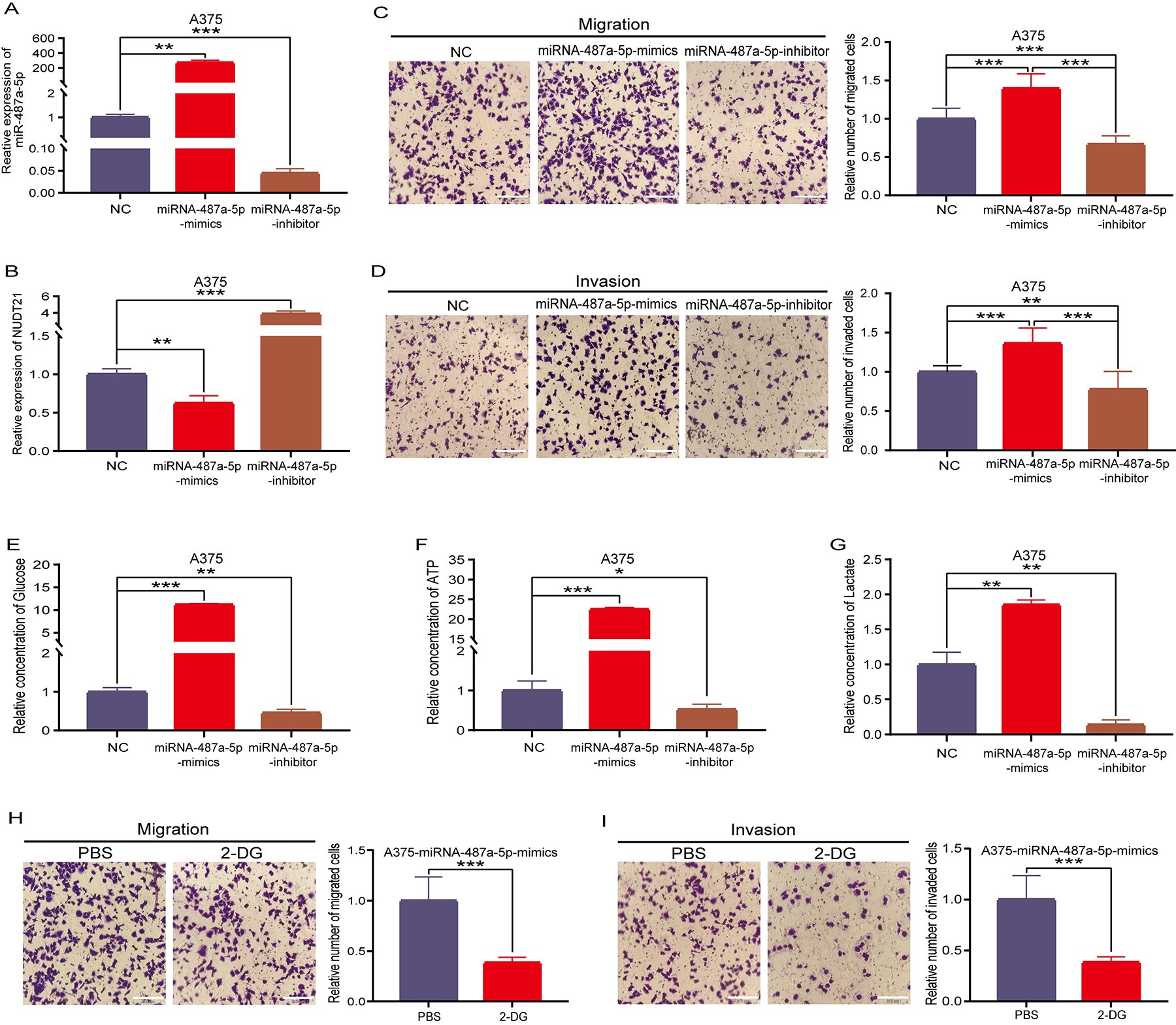

Supplement: Supplementary file 5 — Fig. S5. NUDT21 affects melanoma metastatic competency through glycolytic pathway. (A, B) The expression of (A) miRNA‐487a‐5p and (B) NUDT21 in A375 cells transfected with miRNA‐487a‐5p‐mimics or inhibitor was detected by RT‐qPCR; n = 3. (C, D) miRNA‐487a‐5p‐mimic treatment significantly enhanced the (C) migration and (D) invasion abilities of A375 cells, while A375‐miRNA‐487a‐5p‐inhibitor cells showed weakened invasiveness (bar = 210 μm); n = 3. (E‐G) The contents of (E) glucose, (F) ATP, and (G) lactate were significantly higher in A375‐miRNA‐487a‐5p‐mimics cells, while reduced in A375‐miRNA‐487a‐5p‐inhibitor cells; n = 3. (G, H) 2‐DG (40 μM) inhibited the (G) migration and (H) invasion abilities of A375‐miRNA‐487a‐5p‐mimics cells (bar = 210 μm); n = 3. n = 3: three times a particular experiment was replicated. Error bars indicated SD. *p < 0.05, **p < 0.01, ***p < 0.001 by t‐test. [file MOL2-17-2743-s004.tif]
