# Supplementary material for: Synergistic inhibition of NUDT21 by secretory S100A11 and exosomal miR‐487a‐5p promotes melanoma oligo‐ to poly‐metastatic progression
Source: Mol Oncol. 2023 Jul 1;17(12):2743–66. doi: 10.1002/1878-0261.13480 (PMC10701767; doi:10.1002/1878-0261.13480)
Supplement: Supplementary file 6 — Table S1. The primer sequences. [file MOL2-17-2743-s006.docx]

| **ID** | **5’ to 3’** |
| --- | --- |
| miR-339-5p | TCCCTGTCCTCCAGGAGCTCACG |
| miR-4488 | TATATAGGGGGCGGGCTCC |
| miR-1307-3p | TATACTCGGCGTGGCGTCG |
| miR-374c-3p | GCGCGCACTTAGCAGGTTGTATTATAT |
| miR-412-5p | CGTGGTCGACCAGTTGGAAAGTAAT |
| miR-493-3p | TGAAGGTCTACTGTGTGCCAGG |
| miR-487a-5p | GGTGGTTATCCCTGCTGTGTTCG |
| miR-6529-5p | GAGAGATCAGAGGCGCAGAGTG |
| miR-1246 | CGCGAATGGATTTTTGGAGCAGG |
| miR-383-5p | CGAGATCAGAAGGTGATTGTGGCT |
| miR-197-3p | TTCACCACCTTCTCCACCCAG |
| miR-376b-3p | GCCGCGATCATAGAGGAAAATCCATG |
| miR-331-3p | TATAGCCCCTGGGCCTATCCTAGAA |
| miR-411-5p | CCGCTAGTAGACCGTATAGCGTACG |
| U6 | GCTTCGGCAGCACACATACTAAAAT |

| **ID** | F（5’ to 3’） | R（5’ to 3’） |
| --- | --- | --- |
| Sec23a | AGTGGCGGAAGTCAGGATAC | GGCATTGGAAATCTGGAGTG |
| Nudt21 | CGGCAACAAGTACATCCAGC | AGGGACTGCAAACAAGGCTT |
| Sparc | CCCCTGCCAGAACCATCATT | GCTCAGTGTGGGACAGGTAC |
| Gapdh | TTCACCACCATGGAGAAGGC | TGAAGTCGCAGGAGACAACC |
| S100a11 | AGAGACTGAGAGATGCATTGAG | TATAGCTAAGCCACCAATGAGG |
| Rnf11 | CCATCTATCATCCGACACCTAG | CTTCCAGGGTCATAAACTCCTT |
| Rab31 | TGGGTTTGCAGTGGTTGAGT | CTGTGCCTCACTTATGGGCA |
| Arid4B | GAAGATAACAGCAGCGAAGAAG | AGGCCGTTTGTTAATAGGTGTA |
